# Proliferative regulation of alveolar epithelial type 2 progenitor cells by human *Scnn1d* gene

**DOI:** 10.7150/thno.37023

**Published:** 2019-10-18

**Authors:** Runzhen Zhao, Gibran Ali, Jianjun Chang, Satoshi Komatsu, Yoshikazu Tsukasaki, Hong-Guang Nie, Yongchang Chang, Mo Zhang, Yang Liu, Krishan Jain, Bock-Gie Jung, Buka Samten, Dianhua Jiang, Jiurong Liang, Mitsuo Ikebe, Michael A. Matthay, Hong-Long Ji

**Affiliations:** 1 Department of Cellular and Molecular Biology, 11937 US Hwy 271, University of Texas Health Science Centre at Tyler, Tyler, Texas 75708-3154, USA.; 2 Institute of Health Sciences, Key Laboratory of Medical Cell Biology of Ministry of Education,; 3 Department of Stem Cells and Regenerative Medicine, College of Basic Medical Science, No. 77 Puhe Road, China Medical University, Shenyang North New Area, Shenyang, Liaoning Province 110122, China.; 4 Barrow Neurological Institute, 350 West Thomas Road, St. Joseph's Hospital and Medical Centre, Phoenix, Arizona 85013-4409, USA.; 5 Institute of Lung and Molecular Therapy, 601 Jinsui Avenue, Xinxiang Medical University, Xinxiang, Henan Province 453003, China.; 6 Department of Pulmonary Immunology, 11937 US Hwy 271, University of Texas Health Science Centre at Tyler, Tyler, Texas 75708-3154, USA.; 7 Department of Medicine and Women's Guild Lung Institute, 8700 Beverly Blvd., Suite 6724, Cedars-Sinai Medical Centre, Los Angeles, California 90048-3326, USA.; 8 Department of Medicine and Anesthesia, 505 Parnassus Avenue, University of California San Francisco, San Francisco, California 94143-2204, USA.; 9 Texas Lung Injury Institute, 11937 US Hwy 271, University of Texas Health Science Centre at Tyler, Tyler, Texas 75708-3154, USA.

**Keywords:** alveolar type epithelial cells, epithelial sodium channels, self-renewal, humanized transgenic mouse line

## Abstract

Lung epithelial sodium channel (ENaC) encoded by *Scnn1* genes is essential for maintaining transepithelial salt and fluid homeostasis in the airway and the lung. Compared to α, β, and γ subunits, the role of respiratory δ-ENaC has not been studied *in vivo* due to the lack of animal models.

**Methods**: We characterized full-length human δ_802_-ENaC expressed in both *Xenopus* oocytes and humanized transgenic mice. AT2 proliferation and differentiation in 3D organoids were analysed with FACS and a confocal microscope. Both two-electrode voltage clamp and Ussing chamber systems were applied to digitize δ_802_-ENaC channel activity. Immunoblotting was utilized to analyse δ_802_-ENaC protein. Transcripts of individual ENaC subunits in human lung tissues were quantitated with qPCR.

**Results**: The results indicate that δ_802_-ENaC functions as an amiloride-inhibitable Na^+^ channel. Inhibitory peptide α-13 distinguishes δ_802_- from α-type ENaC channels. Modified proteolysis of γ-ENaC by plasmin and aprotinin did not alter the inhibition of amiloride and α-13 peptide. Expression of δ_802_-ENaC at the apical membrane of respiratory epithelium was detected with biophysical features similar to those of heterologously expressed channels in oocytes. δ_802_-ENaC regulated alveologenesis through facilitating the proliferation of alveolar type 2 epithelial cells.

**Conclusion**: The humanized mouse line conditionally expressing human δ_802_-ENaC is a novel model for studying the expression and function of this protein *in vivo* .

## Introduction

Four subunits (α, β, γ, and δ) of the mammalian epithelial sodium channel (ENaC) have been cloned to date 1-3. α ENaC and splicing δ_1_ subunits form functional homomeric channels individually when expressed in oocytes 1, 4, whereas co-expression with β and γ subunits can amplify the channel activity up to two orders of magnitude 1, 4. The first spliced variant of the human *Scnn1d* gene, encoding δ_1_-ENaC, was cloned in 1995 2. The human *Scnn1d* is a homolog of degenerins (DEG) of *Caenorhabditis elegans*, constituting an ENaC branch of the ENaC/DEG superfamily with other three counterparts (α, β and γ subunits) 5-7. Compared with the other three subunits, δ-ENaC is widely expressed in both non-epithelial (*i.e*., brain, heart, ganglion, placenta, and blood) and epithelial cells (*i.e*., trachea, kidney, pancreas, liver, and stomach) 4, 8. Functionally, classic epithelial αβγ-ENaC channels are a major regulator for maintaining electrolytes and fluid homeostasis in the lung as well as the kidney 9-12. Even though δ-ENaC co-exists with αβγ subunits in numerous cells and tissues 4, 8, and regulates the biophysical features and proteolytic properties of αβγ channels 13-15, the function of δ-ENaC remains uncertain. Surprisingly, *Scnn1d* gene is not expressed in rodents, a major obstacle for *in vivo* functional study 16. The scarcity of *Scnn1d* in rodents may explain the discrepancies observed between mice and humans: α-ENaC deficiency results in the death of new-born mice but not human neonates due to unresolved amniotic fluid in the distal airspaces 17-19. In sharp contrast, the major phenotype associated with a deletion in human chromosome 1, which is composed of the *Scnn1a* gene and others, is growth retardation 20, 21. The expression levels of δ-ENaC is similar to that of α-ENaC in human respiratory epithelial cells, and ~ 40% of amiloride-sensitive sodium transport is associated with δ-ENaC 22-24. Moreover, children with genetic deletion of *Scnn1d* are predisposed to respiratory infection and nasal congestion 25. However, the physiological role of δ-ENaC in normal lungs remains unknown. In addition to the paucity of *Scnn1d* in rodents, the research on *Scnn1d* has long been hindered by lack of pharmaceutical modulators specific for δ-ENaC activity. We have recently cloned a full-length human *Scnn1d* gene (δ_802_-ENaC). Compared with previously identified δ_1_ and δ_2_ splicing variants that are composed of 638 and 704 amino acid (aa) residues, respectively, this δ_802_-ENaC clone encodes 802 aa 4, 8. The aim of this study thus were twofold. First, to test the feasibility of applying α-13 inhibitory peptide to separate α- and δ-type channel populations pharmacologically. α-13 inhibitory peptide is an extracellular segment released by proteolytic cleavage of α-ENaC proteins by proteases 26-28. Second, to characterize the expression and function of human δ_802_-ENaC *in vivo* in a newly established humanized transgenic mouse line in normal animals.

## Results

**Figure 1:**
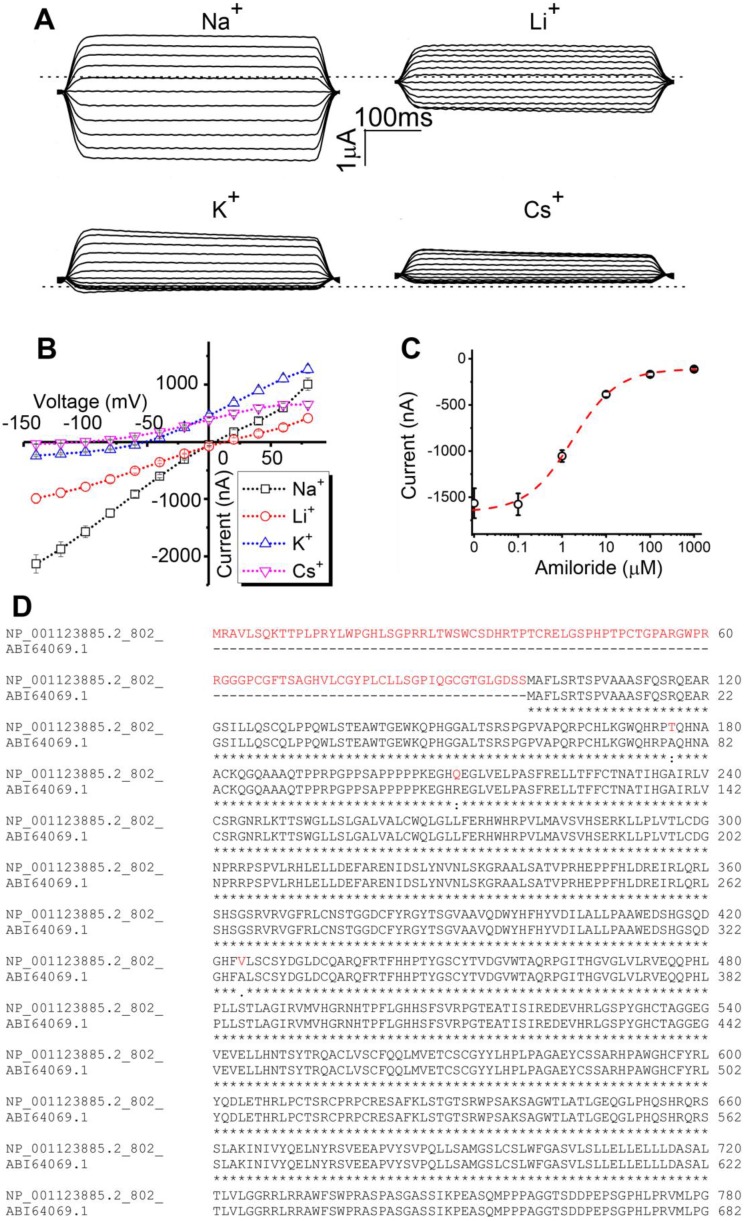
Bioelectric features of full-length human δ_802_ epithelial sodium channels (ENaC) in *Xenopus* oocytes. (A) Representative current trace of human δ_802_βγ ENaC. The channel activity of heterologously expressed δ_802_βγ-ENaC was recorded in cells bathed with Na^+^-, Li^+^-, K^+^-, and Cs^+^-rich bath solutions, respectively. Holding potentials were stepped from -120 mV to +80 mV in an interval of 20 mV. Currents were digitized by the CLAMPEX in the presence and absence of ENaC inhibitor amiloride (10 μM) and then the amiloride-sensitive fractions at each membrane potential were generated with the CLAMPFIT. Dashed line indicates zero current level when the membrane potential was clamped to 0 mV. Scale bars show current level and recording time. (B) Current-voltage relationship of δ_802_βγ-ENaC. Average amiloride-inhibitable currents (Current) were plotted as a function of membrane potentials (Voltage). The reversible potentials are approximate +13 mV for Na^+^ ions, +7 mV for Li^+^ ions, -54 mV for K^+^ ions, and -116 mV for Cs^+^ ions. n=9.** (**C) Dose-response curve for amiloride. Accumulating doses of amiloride were perfused to oocytes expressing δ_802_βγ-ENaC. Current levels at each dose were plotted against applied dose of amiloride and then fitted raw data with the Hill equation to calculate IC_50_ value (1.69 ± 0.3 μM). n=17. Dashed line (red) is generated with the fitted parameters. (D) Comparison of amino acid sequences at the N-terminal tails of δ_802_ (full-length) and δ_2_ clones (previously known as δ_2_-ENaC). Letters in red font show the extended N-terminal tails and three different amino acid residues in the δ_802_ clone only. Data in (B) and (C) are mean ± s.e.m. The data were analyzed using one-way ANOVA followed by Tukey *post hoc* analysis.

**Cloning and characterization of human δ_802_-ENaC in *Xenopus* oocytes**. Two spliced variants of human δ-ENaC have been reported, δ_1_ and δ_2_, which are composed of 638 and 704 amino acid residues, respectively 4, 29. Based on the nucleotide and amino acid alignments of δ_802_ and δ_2_ clones (Figure S1), we extended the N-terminal of δ_2_ clone and substituted a few amino acid residues. As described previously, the cRNA of δ_802_-ENaC was prepared *in vitro* for the heterologous expression in *Xenopus* oocytes 30, 31. Similar to δ_1_ and δ_2_ clones, δ_802_-ENaC was more permeable to Na^+^ ions over Li^+^ ions when co-expressed with the complimentary β and γ subunits (Figure 1A). The order of permeability was Na^+^>Li^+^>K^+^>Cs^+^ ions. A linear chord conductance was observed for predominant permeants Na^+^ and Li^+^ ions (Figure 1B). In contrast, outward currents carried by K^+^ and Cs^+^ ions were greater than those inward charge flows against the K^+^ gradient across the plasma membrane. In agreement with predicted reversal potential by the Nernst equation, the ion selectivity of δ_802_-ENaC acted as a Na^+^ permeable channel. Moreover, amiloride, a specific ENaC inhibitor, suppressed δ_802_-ENaC activity with a *k_i_* value of 1.69 ± 0.3 μM (Figure 1C). The extended N-terminal tail of the δ_802_-ENaC was shown in red font (Figure 1D). These results suggest that heterologously expressed δ_802_βγ-ENaC channels are characterized by Na^+^ selectivity and amiloride inhibition.

**Figure 2:**
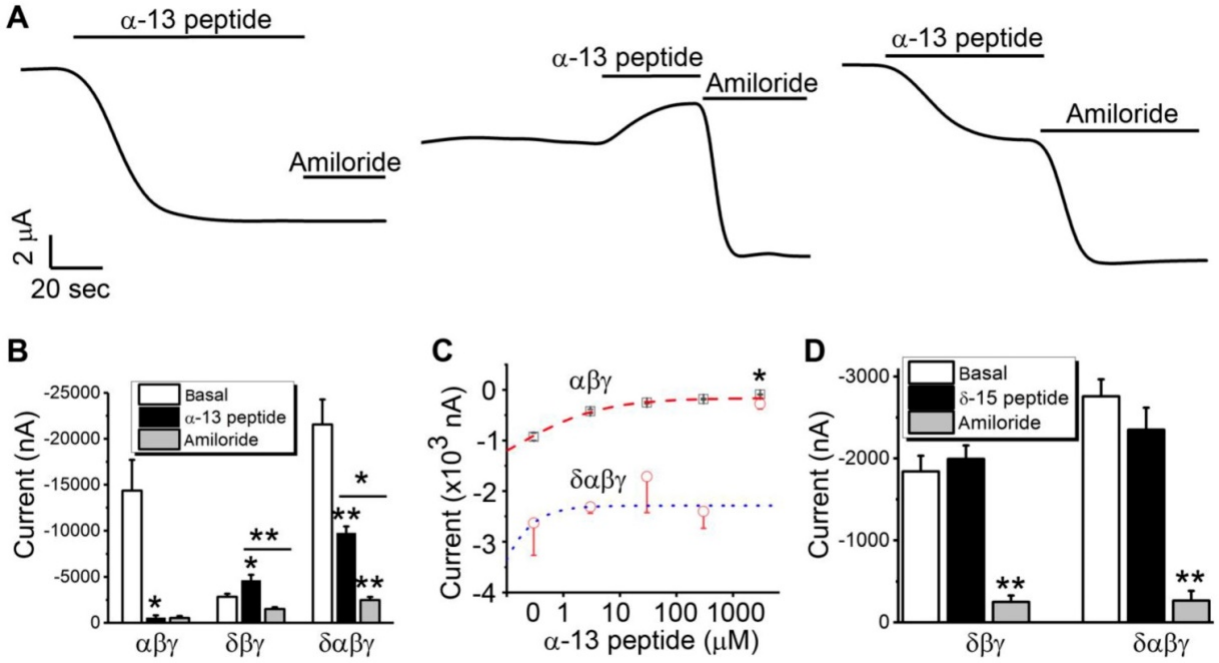
Responses of δ_802_-ENaC-containing channels to α-13 inhibitory peptide. (A) Representative current trace of αβγ- (left), δ_802_βγ- (middle), and δ_802_αβγ-ENaC (right) expressed in *Xenopus* oocytes. The horizontal lines under α-13 (300 μM) and amiloride (10 μM) indicate the time for application. Scale bars at the left bottom corner are for time and current amplitude. The current traces were digitized at the membrane potential of -100 mV. (B) Average current amplitude at -100 mV for the three types of ENaC channels in the absence (basal) and presence of α-13 inhibitory peptide and amiloride. n=5. * P < 0.05 and ** P < 0.01 vs basal current levels or as indicated by the horizontal lines. (C) Concentration-effect relationship of α-13 inhibitory peptide on αβγ-ENaC channels. Dashed (for αβγ) and dotted lines (for δ_802_βγ) are generated by fitting the raw data points except the most right one for amiloride with the Hill equation. The retrieved IC_50_ values for α-13 inhibitory peptide are 0.1 ± 0.01 μM (n=4, Chi^2^ = 0.44, R^2^ = 0.993), and 0.04 ± 0.07 μM (n=3, Chi^2^ = 0.77, R^2^ = 0.86), respectively. * indicates the current levels in the presence of amiloride (10 μM). (D) Effects of δ-15 peptide corresponding to α-13 sequence. δ-15 peptide was designed by aligning the amino acid sequences of δ_802_- and α-ENaC subunits. The sequence of 15 amino acid residues corresponding to that of α-13 inhibitory peptide was synthesized (Figure S2). The same concentration (30 μM) was perfused to the oocytes expressing δ_802_βγ- and δ_802_αβγ-ENaC channels. Amiloride (10 μM) was added to confirm the expression of ENaC. Current data at -100 mV were mean ± s.e.m. ** P<0.01 vs basal levels. n=3.

**Distinguishing αβγ and δ_802_βγ-ENaC sub-populations functionally with the combination of α-13 peptide and amiloride**. Autoinhibitory peptides proteolytically released from α- and γ-ENaC subunits functionally block the heterologously expressed mouse and human αβγ-ENaC activity 26, 32-34. However, it is unknown if these peptides alter the δ-containing channels. Given the physical and functional subunit-subunit interactions between four ENaC subunits and the heterotrimeric 3D model of human ENaC protein complexes 35, inhibitory peptide released from γ-ENaC may block both αβγ- and δβγ-type channels. Thus, we attempted to test if α-13 peptide (Figure S2) released from α-ENaC post proteolysis inhibits αβγ- but not δ_802_βγ-ENaC channels. As shown in Figure 2A, the α-13 peptide (300 μM) has the same efficacy as amiloride (10 μM) to inhibit the human αβγ-ENaC, since subsequent addition of amiloride did not reduce channel activity further. In sharp contrast, the δ_802_βγ-ENaC activity was slightly elevated by the α-13 peptide without statistical significance. All four ENaC subunits are co-expressed in many tissues 29, 36, 37.

Approximately half of the total activity associated with δαβγ-ENaC channels could be suppressed by α-13 peptide, whereas approximately 80% of the α-13 peptide-resistant remaining fraction of current amplitude was inhibited by amiloride (Figure 2B). Over expression of δ_802_-ENaC increased the fraction of the amiloride-resistant current (Figure 2B), and improved the sensitivity of αβγ channels to α-13 peptide (*k_i_*: 0.1 ± 0.01 μM for αβγ vs 0.04 ± 0.07 μM for δ_802_αβγ) (Figure 2C). Furthermore, we tested the potential effects of a peptide in δ_802_-ENaC (δ-15 peptide, Figure S2) analogous to α-13 peptide sequence (Figure 2D). This peptide did not significantly affect the activity associated with both δ_802_βγ and δ_802_αβγ channels. These data show that α-13 peptide can be used to separate native αβγ- and δβγ-ENaC channel activities in epithelial tissues.

**Proteolytic activity of δ_802_-containing channels**. γ-ENaC can be proteolytically cleaved by serine proteases 33, 38, 39, which can be facilitated by δ_1_-ENaC co-expression in oocytes 14.

**Figure 3:**
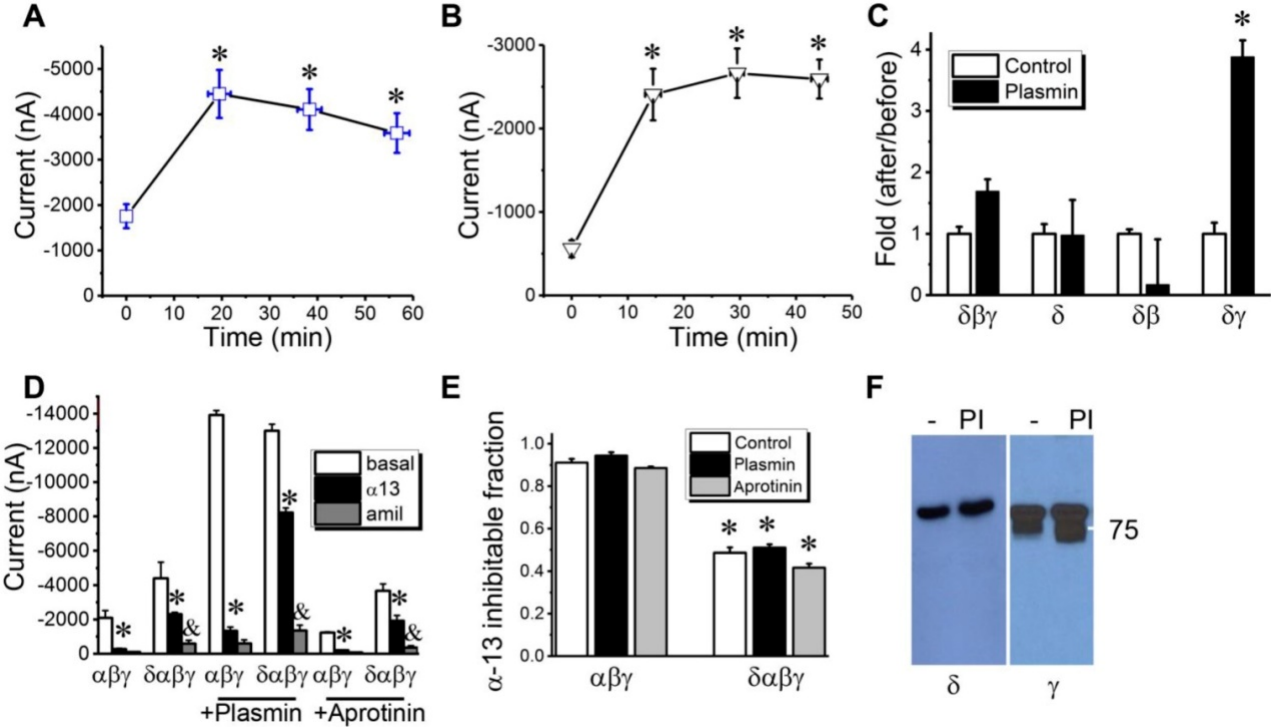
Activation of δ_802_-containing ENaC channels by serine proteases. (A) Activation of δ_802_βγ ENaC expressed in *Xenopus* oocytes by two-chain urokinase plasminogen activator (tc-uPA). ENaC activity was measured at the membrane potential of -100 mV at defined time points post incubation with 10 μg/ml tc-uPA. n = 9-15. mean ± s.e.m. * P<0.05 vs data at the zero minute (min). (B) Time course of stimulatory effects of plasmin on δ_802_βγ-ENaC activity. n=12. * P<0.05. (C) Identification of subunit responsible for plasmin activation. Fold of amiloride-sensitive current (ASI) of ENaC-associated current in the presence of plasmin over that in the absence of plasmin was computed for oocytes expressing δ_802_+β+γ (δβγ), δ_802_ alone (δ), δ_802_+β(δβ), and δ_802_+γ (δγ). n = 12-15. * P<0.05. (D) Inhibitory effects of α-13 peptide on δ_802_αβγ- (δαβγ) and αβγ-ENaC activity post treatment of plasmin and aprotinin. Currents (I) at -100 mV were compared in the absence of amiloride (basal, open bar), presence of α-13 peptide (α13, closed bar), and amiloride (amil, grey bar). n=5. * P<0.05 vs basal currents, & P < 0.05 vs those in the presence of α-13 peptide. (E) Fractions of α-13 sensitive currents in cells pre-treated with plasmin and aprotinin. n=8. Control, without pre-treatment. (F) Cleavage of V5-tagged δ_802_- and γ-ENaC subunits by serine proteases. NI, non-injected oocytes as negative controls for ENaC expression. Oocytes incubated with chymotrypsin (CT, 10 μg/ml), plasmin (PI, 10 μg/ml) for 45 minutes at room temperature. Cells treated with the same volume of saline (‑) were controls for serine proteases.

Whether the full-length δ_802_-ENaC with extended N-terminal is catalyzed by a serine protease is uncertain. We thus incubated the oocytes expressing δ_802_βγ subunits with serine proteases. Two-chain urokinase plasminogen activator (tc-uPA) (Figure 3A) and plasmin (Figure 3B), two crucial molecules of the plasmin(ogen) signal pathway for fibrinolysis, activated the δ_802_βγ activity in minutes. Next, the results showed that γ-ENaC subunit could be a target of plasmin (Figure 3C). The activation of δ_802_βγ-ENaC activity could be up and down regulated by β- and γ-ENaC subunits, respectively. To test whether the fibrinolytic activity alters the blockade of α-13 peptide on ENaC channels, oocytes were pre-treated with plasmin and antiprotease aprotinin (Figure 3D). Plasmin potentiated both αβγ- and δ_802_αβγ-ENaC activities significantly to a similar level. On the other hand, aprotinin did not alter the current amplitude significantly. Thus, the α-13 inhibitable fraction of the αβγ-ENaC channels was not significantly affected by fibrinolytic activity (Figure 3E), suggesting that α-13 peptide may not be cleaved by plasmin, and that the binding site of α-13 peptide may not be altered by the proteolysis-induced structural changes in ENaC protein complexes. Similarly, the activity of δ_802_αβγ channels was inhibited by α-13 peptide to approximately 50% of the total current magnitude in the presence and absence of plasmin and aprotinin. To examine the cleavage of ENaC proteins by plasmin, we incubated oocytes co-expressing δ_802_- (HA- and His-tagged) and γ-ENaC (HA- and V5-tagged) with complementary subunits with either chymotrypsin or plasmin (Figure 3F). Neither chymotrypsin nor plasmin nor endogenous furin cleaved the δ_802_ subunit. In striking contrast, γ-ENaC was catalyzed by furin-like protease and further by plasmin as shown with the cleaved band(s) in different sizes. These results suggest that activation of δ_802_βγ-ENaC channels may be due to the cleavage of γ-ENaC by plasmin. Moreover, both α-13 peptide *per se* and its inhibitory effects on the ENaC function were not altered by fibrinolytic activity, which is most frequently cleaves the γ subunit.

**Figure 4:**
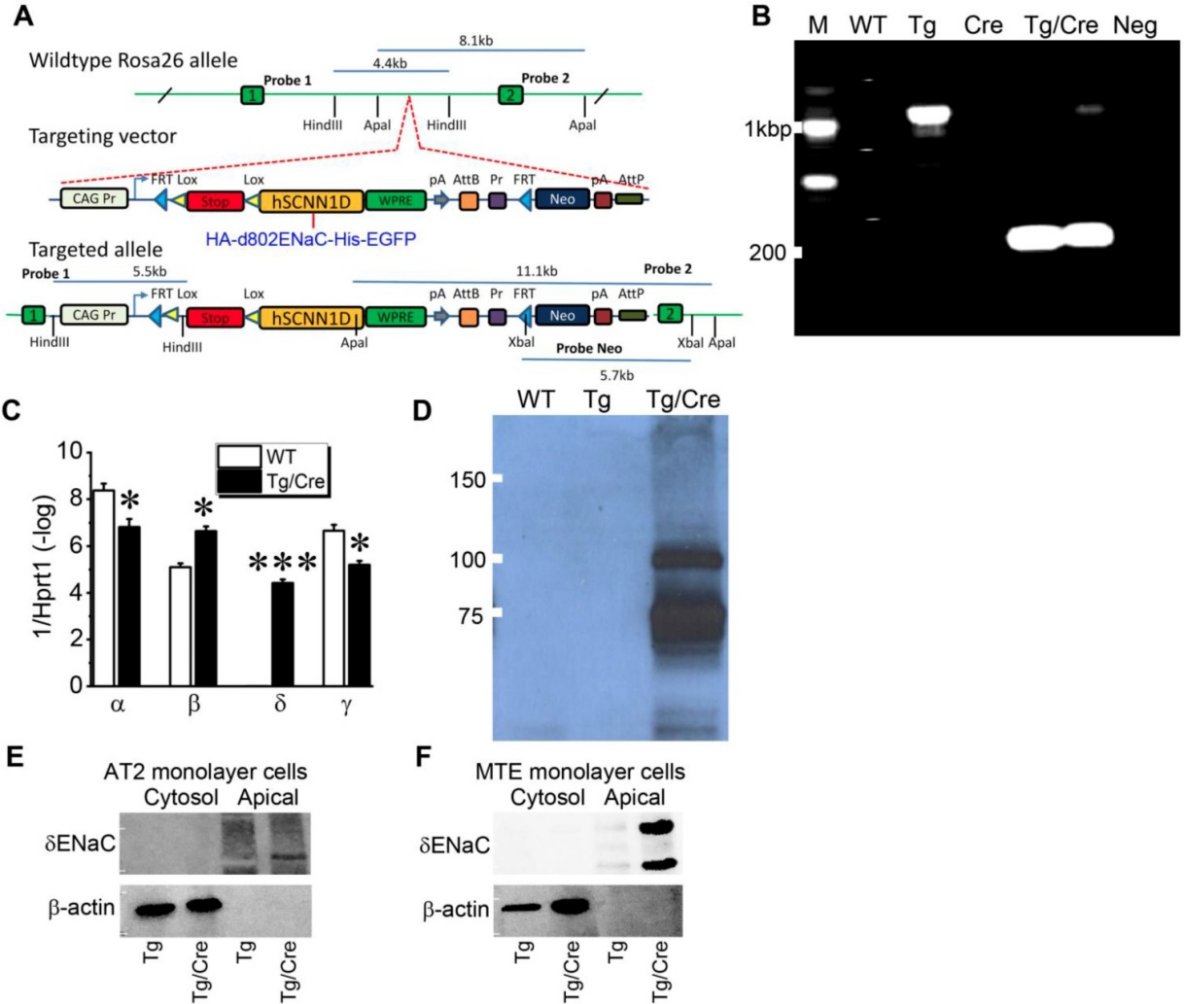
Humanized mouse line with inducible expression of human δ_802_-ENaC. (A) Schematic design for developing humanized mouse strain. δ_802_-ENaC was labelled with epitope tags of HA at the N-terminal and His at the C-terminal ends. The tagged hSCNN1D construct (HA-δ_802_ ENaC-His) was inserted into Rosa26 allele. Two lox and one stop codon were placed just prior to this construct. (B) RT-PCR analysis of inducible expression of δ_802_-ENaC. 3× stop primers were used with a size of 1,070 bp. The size is 199bp after removal of 3× stop codons. From left to right are samples from wild type lungs, *Scnn1d* Tg lungs, sox2 Cre lungs, δ_802_^Tg/Cre^ lungs, and a negative control in the absence of RT enzyme. (C) Transcripts of ENaC subunits in the lung. The mRNA level of four ENaC subunits was analyzed by real-time RT-PCR. * P<0.05 and *** P<0.001 vs wt control. n=3. (D) Immunofluorescent images of δ_802_ ENaC proteins in the lung. Lung sections were stained with DAPI (blue for nuclei) and anti-His tag antibody to recognize δ_802_ ENaC (red). Scale bar, 10 μm. From left to right are images for wild type (WT, left), *Scnn1d* Tg (Tg, middle), and δ_802_^Tg/Cre^ lungs (Tg/Cre, right). Top panels are for alveolar type 1 (AT1) cells stained with AQP5 as a biomarker. Bottom panels are for alveolar type 2 (AT2) cells stained with sftpc as a biomarker. (E) Detection of δ_802_ ENaC expression at the protein level with Western blotting assays. Tissue lysates of wt, *Scnn1d* (Tg), and induced lungs (Tg/Cre) were probed with anti-HA antibody. (F) Immunoblotting biotinylated apical proteins recognized by anti-HA antibody. Top blots are for δ-ENaC in apical and cytosolic proteins. β-actin was used as loading controls and quality control for biotinylation. These blots represent three experiments with similar results.

**Inducible expression of human *Scnn1d* in mouse airway and lung epithelium**. In contrast to human and other species, mice do not express *Scnn1d* gene 8, 36. To overcome this barrier for studying human δ-ENaC *in vivo* , we developed a humanized inducible transgenic mouse strain expressing newly cloned full-length human δ_802_-ENaC (Figure S3). Because commercially available antibodies against human δ_802_-ENaC have not systematically characterized *in vivo* and potential post-translational modifications may modify the 3D structure to dissect or cover the antigens, we attached HA and His tags to the N- and C-termini of the δ_802_-ENaC, respectively. The tagged construct was then inserted into EGFP-tagged ROSA 26 allele post the stop codon (Figure 4A). Sox2^Cre^ was cross-bred with the humanized δ_802_^Tg^ to express human δ_802_-ENaC globally to mimic the expression pattern in human tissues. Using the primers for the stop codon, we detected a PCR product of approximate 200 bp only in induced lungs (Figure 4B). As detected with qPCR with the primers for ENaC subunits, the expression of human δ_802_-ENaC in mouse lungs was only found in the induced animals (Figure 4C). Comparing to the wt controls, the native α- and γ-ENaC transcripts were reduced slightly but β subunit was transcribed to a greater extent post induction of the human *Scnn1d* expression. These observations were further corroborated by the expression of δ_802_-ENaC proteins in whole lung lysates by Western blot (Figure 4D), with a size of 87.85 kDa band as predicted and a larger band at about 110 kDa. Both bands were confirmed as human δ_802_-ENaC proteins with mass spectrometry (Figure S4). To locate the subcellular expression of δ_802_-ENaC in the apical membrane of the airway and lung epithelial cells, we grew primary mouse AT2 cells and mouse tracheal epithelial (MTE) cells as polarized monolayers. The results show that δ_802_-ENaC was localized at the apical membrane as recognized by anti-HA antibody in biotinylated apical proteins from both primary mAT2 monolayer cells (Figure 4E) and MTE cells (Figure 4F). In addition, the band at 110 kDa was only found in polarized MTE cells. These studies demonstrate the expression of δ_802_ at the apical membrane of the respiratory epithelium *in vivo* .

**Figure 5:**
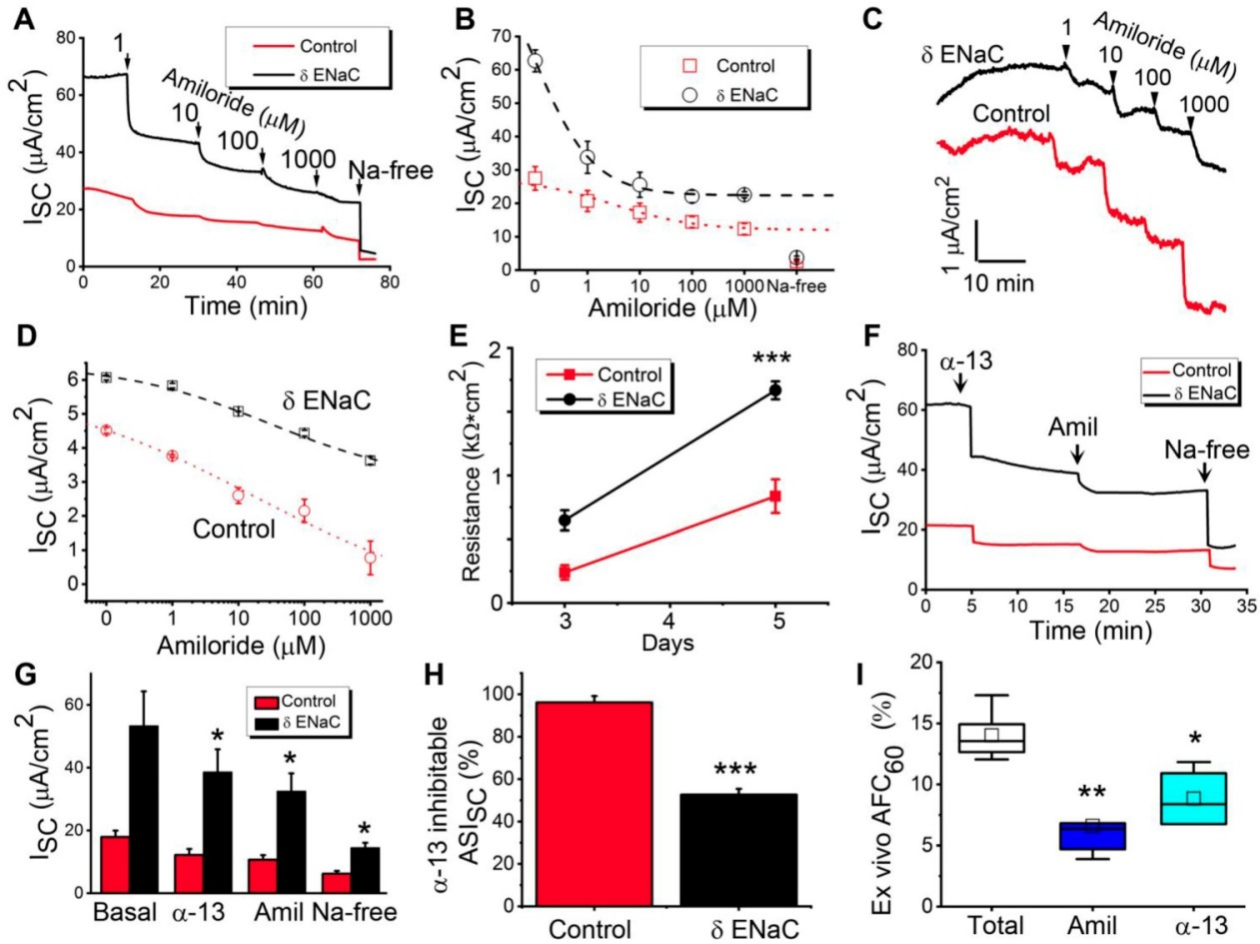
Functional analysis of δ_802_ ENaC activity in mouse tracheal epithelial (MTE) and alveolar type 2 (mAT2) monolayers. (A) Short-circuit current traces (Isc) in MTE monolayers mounted on an Ussing chamber setup for uninduced δ_802_^Tg^ (Control, black line) and induced δ_802_^Tg/Cre^ (δ-ENaC, red line). Primary MTE cells were cultured at the air-liquid interface for 13-15 days. n=3. Arrows indicate the time to add designed concentrations of amiloride. (B) Dose-response relationship of amiloride for δ-ENaC expressing MTE monolayers and controls. Raw data were fitted with the logistic function to compute *k_i_* values for amiloride. The resulting *k_i_* values were 0.68 ± 0.00 μM and 3.04 ± 0.26 μM, respectively, for control and δ-ENaC group. Data collected when cells were bathed with Na^+^-free solution were included but not used for fitting curves. n=5. (C) Representative Isc traces for control and δ-ENaC mAT2 monolayer cells. Accumulating amiloride from 1 to 1,000 μM were applied as indicated by arrows. (D) Dose-response curve for amiloride in mAT2 cells. (E) Transepithelial resistance measured with an EVOM meter. Resistance was read with a chopstick meter when the culture medium was replaced every other day. n=7, *** P<0.001 vs controls. (F) Representative Isc traces for α-13 peptide inhibition. α-13 inhibitory peptide (300 μM) was added to the apical counterpart followed by amiloride and finally by Na^+^-free bath solution as indicated. (G) Average Isc levels in the presence and absence of α-13 inhibitory peptide (300 μM), amiloride (100 μM), and Na^+^ ions (150 mM). n=6. * P<0.05 vs control. (H) Isc fractions associated with αβγ-ENaC and δ_802_βγ-ENaC subpopulations as computed as α-13 inhibitable (α-13 peptide sensitive) and the component inhibited by amiloride and removal of bath Na^+^ ions (α-13 peptide resistant). n=6. * P<0.05 vs controls. (I) α-13 and amiloride-sensitive alveolar fluid clearance in human lungs *ex vivo*. n=5 per group. * P<0.5 vs control. Data were presented as mean ± s.e.m., mean difference between two groups was computed by student t-test in (E-H).

**Bioelectric features of δ_802_-ENaC channels in primary MTE and AT2 monolayer cells**. To characterize the function of human δ_802_-ENaC expressed in the airway and alveolar epithelial cells, we harvested primary MTE and AT2 cells to grow polarized tight monolayers at the air-liquid interface (Figure S5**)**. The basal transepithelial short-circuit current (Isc) level of MTE monolayers was greater for δ_802_-ENaC group over the controls (Figure 5A). The Isc currents in both control and δ_802_ ENaC monolayers were gradually inhibited by accumulating concentrations of amiloride (1 - 1,000 μM) and eliminated by removing Na^+^ ions from the bath solutions. The blockade of the ENaC activity in monolayers by amiloride was concentration-dependent with a greater apparent *k_i_* value for δ_802_-ENaC group (Figure 5B). Furthermore, we repeated these observations in the AT2 monolayers (Figure 5C). Like those in the MTE cells, expression of the human δ_802_-ENaC in AT2 cells elevated Isc amplitude significantly. In both MTE and AT2 monolayers, the apparent *k_i_* value of amiloride was much lesser in controls than that in the δ_802_-expressing cells (Figure 5D). We measured monolayer resistance and a significant difference was measured between controls and δ_802_-expressing AT2 cells (Figure 5E). The results in oocytes (Figure 2) suggest that α-13 inhibitory peptide may be used to distinguish native α-type and human δ-containing ENaC subpopulations. To test this hypothesis, we applied the α-13 autoinhibitory peptide to the apical counterpart followed by amiloride and then by Na^+^-free bath solution in MTE monolayers (Figure 5F). As shown in Figure 5G, Isc values were suppressed to a different extent between control and δ_802_-ENaC group by α-13 peptide, amiloride, and Na^+^ ion depletion. α-13 peptide-inhibitable fraction of Isc was 96% and 52%, respectively, for control and δ_802_-ENaC cells (Figure 5H). Finally, we confirmed these observations by measuring *ex vivo* alveolar fluid clearance in ex vivo human lungs (Figure 5I). Approximately half of the total alveolar fluid clearance was inhibited by α-13 peptides, suggesting that there are at least two ENaC sub-populations (i.e., α-type and δ-type) in human lungs, and that the subsequent application of α-13 peptide (αβγ-ENaC) and amiloride (both αβγ and δβγ channels) can separate them functionally *in vitro* and *in vivo* .

**Figure 6:**
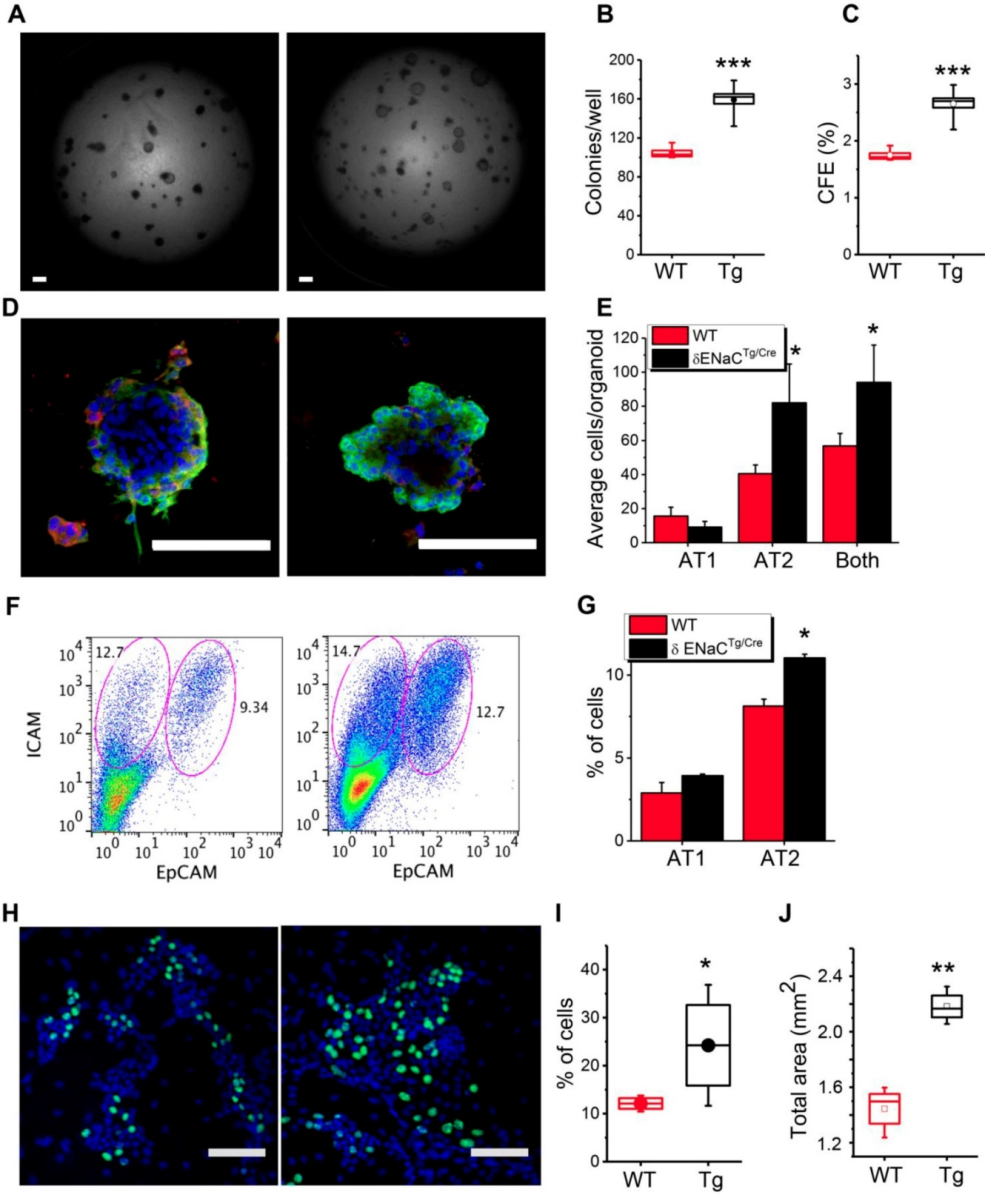
Contribution of δ_802_-ENaC to progenitor mAT2 cell-mediated alveologenesis. (A) DIC images of mAT2 organoids. Primary mAT2 cells of δ_802_^Tg^ (left) and δ_802_^Tg/Cre^ mice (right) were grown in 3D Matrigel for 7 days. Scale bar, 1 mm. (B) Organoid number per well. n=6. *** P<0.001. (C) Colony forming efficiency (CFE) of mAT2 cells. n=12. *** P<0.001. (D) Confocal imagines of organoids formed by mAT2 from δ_802_^Tg^ (left) and δ_802_^Tg/Cre^ mice. Scale bar, 100 μm. Organoids were stained with DAPI (blue), PDPN antibody for AT1 cells (red), and anti-sftpc antibody for AT2 cells (green). (E) AT2 renewal and differentiation into AT1 cells. n=6 organoids. * P<0.05 vs WT group. (F) FACS assay of AT1 and AT2 cells in organoids. Representative FACS analysis of AT1 (ICAM^+^) and AT2 (EpCAM^+^) cells for WT (left) and δ_802_^Tg/Cre^ group (right). n=3. (G) % of AT1 and 2 cells. n=6. * P<0.05. (H) EdU stain of mAT2 monolayers for WT (left) and δ_802_^Tg/Cre^ groups (right). Polarized mAT2 monolayers on day 7 post seeding were stained with EdU (green). (I) % of EdU^+^ cells. n=6 monolayers per group. * P<0.05. Student t-test. (J) Total surface area of organoids per well. n=6. ** P<0.01.

**δ_802_-ENaC up regulated alveologenesis *in vitro***. ENaC is expressed in pulmonary epithelial stem and progenitor cells and implicated in the repair of injured epithelial and other tissues 40-44. We deciphered the contribution of δ_802_-ENaC in mouse progenitor AT2 cells to alveologenesis in 3D organotypic cultures (Figure 6A). Organoids were imaged under DIC and confocal microscopes (Figure S6**)**. A greater number of alveolar-like organoids was observed in the δ_802_-expressing AT2 cells compared to the controls (Figure 6B), indicating an improved efficiency to generate hollow spheroids (Figure 6C). Moreover, AT1 and AT2 cells were tracked with their specific markers pdpn and sftpc, respectively, to evaluate proliferation and differentiation in individual organoid (Figure 6D). Intriguingly, δ_802_-ENaC augmented the efficiency of AT2 renewal significantly (Figure 6E). This observation was further confirmed by sorting AT1 (ICAM^+^) and AT2 (EpCAM^+^) cells by FACS (Figure 6F-G). In addition, we performed EdU incorporation assays in mouse AT2 monolayers (Figure 6H). More EdU positive AT2 cells were found in cells expressing human *Scnn1d* over those of the wt controls (Figure 6I). The clinical relevance of generating more alveoli by δ_802_-ENaC expression may be to re-epithelialize a larger luminal surface area than controls (Figure 6J).

## Discussion

The main findings of this study can be summarized as follows. We established a novel humanized transgenic mouse model conditionally expressing full-length human *Scnn1d* gene. We found that the *Scnn1d* gene is a regulator of alveolar progenitor AT2 cells and also functions as a critical transepithelial sodium transport pathway.

Like the previous cloned two splicing variants, the δ_802_-ENaC functions as a Na^+^ selective amiloride-inhibitable Na^+^ channel, because the selectivity filter and amiloride-binding sites are localized in the second transmembrane domain. The selectivity filter of δ‐ENaC, as predicted by *in silico* analysis, is composed of G-A-S vertically. By comparison, the most common motifs across species for α-, β-, and γ-ENaC are G-S-S, G-G-S, and S-C-S, respectively 45, 46. Interestingly, the selectivity filter for ASIC channels is also G-A-S with a greater Na^+^ permeability over Li^+^ ions, and both δ_1_βγ and δ_2_βγ channels are activated by external protons. The model of ion selectivity filter is composed of three layers vertically 45, 46. The second layer is different between αβγ (S-G-C) and δβγ-ENaC (A-G-C) channels. In addition, divergent second layers for FaNaC, PPK, RPK, MEC-4, UNC-8, and BLINaC of the ENaC/DEG family are reported 9. This could cause the diverse P_Na_/P_Li_ ratio for δ_802_-ENaC (1.6 vs 0.6 for αβγ ENaC) as evidenced in ASICs, MEC-4, RPK, and BLINaC 9, 47, 48. However, it has not been confirmed by mutagenesis whether the second variant layer of the δ_802_-ENaC is critical for the selectivity of Na^+^ and Li^+^ ions.

Although δβγ-ENaC is less sensitive to amiloride compared with αβγ channels as reported previously 2, 24, the study of δ-type ENaC function, in particular for native δ-containing channels has been limited by the paucity of specific pharmacological modulators 49. Our data provide a novel strategy to separate αβγ and δβγ ENaC activities by subsequently applying α-13 inhibitory peptide and amiloride. This novel strategy can be used for both normal and diseased lungs as the efficacy of α-13 peptide is not influenced by abnormal fibrinolytic activity. In addition, serine protease and α-13 peptide target γ- and α-ENaC subunits, respectively. Because α-13 peptide and amiloride have different binding sites and subunit-specific for α-13 peptide, there may not be an intermolecular regulation between them.

Our data support the hypothesis that δ-ENaC can benefit the alveolarization by re-epithelializing greater area of alveolar epithelial layer. The potential mechanisms are related to the augmented proliferation of alveolar progenitor mouse AT2 cells. Up regulation of ENaC activity by steroids, CPT-cGMP, miRNAs, and fluid flow improves stem/progenitor cell proliferation *in vitro* and *in vivo* 43, 44, 50, 51. In contrast, suppression of β-ENaC expression reduces cell proliferation significantly 52. Apparently, both expression and channel activity of δ-ENaC may contribute to the increased proliferation of mAT2 cells in generating alveolus-like spheroids. In addition, increased DNA synthesis in *Scnn1d* cells could be associated with AT2 proliferation. An interesting topic for future research would be to characterize potential *Scnn1d*/DNA synthesis/proliferation/alveologenesis cascade. On the other hand, we cannot exclude the potential contribution of *Scnn1a*, *Scnn1b*, and *Scnn1g* to the fate of AT2 cells. Broadly speaking, the linage of AT2 cells could be regulated by more than one gene. To the best of our knowledge, this is the first study on the regulation of AT2 fate by *Scnn1d* genes.

The effects of the extended cytosolic NH2-terminal tail for the δ_802_-ENaC are unknown. The N-terminal tails of α, β, and γ-ENaC subunits are involved in endocytosis-related channel activity 53, 54, PIP2 binding and hydrolysis 55, 56, intracellular gating 57, 58, interaction with CFTR 31, and proteolysis 14, 59, 60. By comparing of δ_1_βγ and δ_2_βγ channels in oocytes, it was found that δ_2_ channels had an increased single channel conductance and activity 29. The differences in apparent Na^+^ affinity, amiloride sensitivity, and activation by capsazepine and external protons were also observed 29, which could be attributed to their differentiated trafficking 61.

Whether the effects of δ_802_-ENaC on AT2 proliferation could be recapitulated *in vivo* for homeostasis and regeneration remains obscure. We did not see the significant difference in the yield of AT2 cells between adult healthy *Sox2*^cre^ and *Scnn1d*^Tg/cre^ mice. These could be due to considerable loss of AT2 cells during isolation and the difference in niche for AT2 cells between *in vivo* and *in vitro* . The scenario for alveologenesis in fetal lungs and regeneration of the air sacs in injured lungs could fit the *in vitro* 3D cultures.

Although there is a considerable decrease in AT1 cells (pdpn positive) in *Scnn1d*^Tg/cre^ organoids, as counted by the ImageJ Cell Count Plug-in, the reduction was not seen when total detached cells from all organoids in each Transwell were analyzed by FACS (ICAM positive) (**Fig 6f-g**). Technically, this discrepancy could be resulted from the unidentical affinity between pdpn and ICAM antibodies. We could not use the same antibody, either pdpn or ICAM, for both confocal imaging and flow cytometry for their diverse applications.

Whether the contribution of delta-ENaC to alveolar fluid clearance is mediated by its proliferative effects on AT2 cells is unclear. Considering that the uncertain life time and solubility of α-13 peptide within the Matrigel for up to 9 days, and the broad effects of amiloride on gene expression 36, other transport function (Na^+^/H^+^ exchanger, Na^+^/Ca^2+^ antiporter, Na^+^/Li^+^ exchanger, Na^+^/K^+^-ATPase, etc.) 62, and anti-fibrinolytic activity (inhibitors of uPA and plasmin) 63, the results would be much less rigor due to these uncontrollable variables. In addition, the penetration of amiloride and α-13 peptide through the Matrigel is unknown if applied to the culture medium and replaced every other day. Therefore, we simply compared the difference in alveologenesis, AT2 proliferation, and channel activity between wt and *Scnn1d*^Tg/cre^ cells in parallel. This well-controlled strategy provide rigorous data.

The clinical implications of Scnn1d in airway fluid homeostasis is emerging. Two mutation variants (V541L and P579L) of Scnn1d in three homozygous F508del males were identified recently 64. They acquired *P. aeruginosa* and were pancreatic insufficient as diagnosed for essential hypertension, nasal polyps, and cystic fibrosis-related diabetes. δ_1(V541L)_βγ mutant when expressed in *Xenopus* oocytes exhibited reduced channel activity. δ_1_βγ channel activity was inhibited by CFTR when co-expressed in oocytes 65, suggesting that loss of down-regulation of *Scnn1d* encoded ion channel activity by deficient CFTR could lead to a hyperactive δ802 channel to exacerbate dehydration of the airways. In addition to the function of *Scnn1d* in human nasal epithelium 22, we for the first time confirmed the contribution of *Scnn1d* gene to alveolar fluid clearance. Taken together, δ-ENaC may function as a critical pathway for apical fluid and electrolyte homeostasis in normal lungs.

In summary, we identified a novel role of human δ-ENaC in lung epithelial cells in a newly developed humanized mouse colony. This mouse model together with the novel combination of α-13 peptide and amiloride may pave the way for a new path for investigating the physiological roles of human δ-ENaC *in vivo* .

## Methods

**Human AT2 cell isolations**. Human AT2 cells were isolated with a modified protocol 66, 67. Briefly, distal lung tissue was obtained and dissected into rough 5 cm^3^ pieces. Tissues were washed in 500 ml sterile PBS for 10 minutes at 4°C at least two times, or until PBS no longer appeared bloody. An additional 10 minutes wash was then performed with Hank's buffered saline solution (HBSS). Tissue was compressed with autoclaved Kim wipes to remove as much liquid as possible and further dissected into 1 cm^3^ pieces. Sterile HBSS buffer containing 5 units/ml dispase II and 0.1 mg/ml DNase I + penicillin/streptomycin was added to the small tissue pieces. The digest solution at this point was rapidly taken up by the tissues, becoming visibly engorged, and was digested 2 hours at 37°C. Fungizone (1:400) was added for the final 30 minutes of the digest. The digest solution was then stored overnight at 4°C without further degradation of cells due to lack of dispase activity at this temperature. The digested tissue was warmed to 37°C and liquefied with an Osterizer 12 speed blender as follows: 5 sec pulse, 5 sec grate, and 2-5 sec pulse. The suspension was poured through a glass funnel lined with sterile 4 × 4 gauze, applying some compression in order to recover as much of the solution as possible. The cell suspension was sequentially filtered through 100 μm, 70 μm, and 40 μm cell strainers. Finally, red blood cells were removed using the red blood cell lysis buffer (Sigma-Aldrich). Antibodies against human CD31, CD45, and EpCAM were purchased from BioLegend (San Diego, CA). Flow cytometry was performed using a FACSCanto II flow cytometer and FACSAria III sorter (BD Immunocytometry Systems, San Jose, CA) and analyzed using Flow Jo 9.6.4 software (Tree Star, Ashland, OR).

**Alveolar fluid clearance in human lungs *ex vivo*.** The studies followed the guide of the Declaration of the People's Republic of China and were approved by the Ethics Committee of the China Medical University (CMU) at Shenyang, China. All patients were given oral and written informed consent forms. Human lung tissues were obtained during pulmonary resection surgeries with lung cancer at the First Affiliated Hospital of CMU. There were no fibrous or emphysematous lesions as assessed by preoperative chest CT scanning. Human lung segments were prepared, and AFC measurements were done as described previously 68. Briefly, the segmental bronchus was occluded by a 10-Fr. balloon catheter immediately after removal of the lung, and occluded segments that were located furthest away from the focus of tumor were chosen. A warmed physiologic saline solution (20 ml; 37°C) containing 5% BSA with or without amiloride (1 mM) was instilled into the distal air spaces through the catheter. After instillation, the lungs were inflated with 100% oxygen at an airway pressure of 7 cm H_2_O. Alveolar fluid was aspirated 60 minutes after instillation. Aspirated alveolar fluid was centrifuged at 3,000 × g for 10 minutes, and the supernatant was obtained for measurement of protein concentrations. AFC values were calculated as follows 68: AFC = [(V_i_ - V_f_)/V_i_] × 100, where V is the volume of the instilled albumin solution (i) and the final alveolar fluid (f). V_f_ = V_i_ × P_i_/P_f_, where P is the concentration of protein in the instilled albumin solution (i) and the final alveolar fluid (f).

***In vivo* alveolar fluid clearance (AFC) in mice***. In vivo* AFC rate was measured as previously described 68-70. Briefly, mice were placed on a continuous positive airway pressure system delivering 100% O_2_ at 8 cmH_2_O. All animals were maintained at a temperature of 37ºC with a heating pad and ultrared bulb. An isosmotic instillate containing 5% bovine serum albumin (BSA) was prepared with saline. To maximize the collection of instilled BSA solution, the diaphragm was dissected. Methodological concerns were addressed by using amiloride to inhibit water movement to confirm that measurements accurately reflected AFC. The endogenous murine albumin would lead to an overestimation of AFC rates. This potential bias was corrected by measuring murine albumin (AssayPro, St. Charles, MO) and bovine albumin (Bethyl Laboratories, Montgomery, TX) separately using specific ELISAs following the manufacturer's instructions 69. Final AFC values were corrected removing murine albumin present in the aspirate. In addition, we used an aliquot of the instillate that had been delivered into the lungs and removed within 5 min, rather than freshly prepared naive instillate *per se*. Any pre-existing edema or leaking murine plasma proteins would be excluded for it dilutes both the control at time point 0 min (*P_i_*) and final samples (*P_f_*). AFC rates were calculated as aforementioned for human lung AFC *ex vivo*.

**Mouse AT2 cell isolation and monolayer cultures**. Mouse AT2 cells were isolated from both wt and *Scnn1d* Tg/Cre strains with C57BL/6 genetic background with a modified protocol of a previous publication 71, 72. Briefly, lungs of euthanized mice were removed and incubated in dispase for 45 minutes, and then were gently teased and incubated in DMEM/F-12 + 0.01% DNase I for 10 minutes. Cells were passed through a serial of Nitex filters (100, 40, 30, and 10 microns; Corning, USA) and centrifuged at 300 × g for 10 minutes. Resuspended cells were incubated with biotin conjugated anti-CD16/32, CD45 and CD119 antibodies (BD Pharmingen, USA) and then incubated with streptavidin-coated magnetic particles. The cells were then incubated for 2 h at 37ºC in a plastic culture dish precoated with mouse IgG to remove fibroblasts. Upon passing the request for both viability (> 90%) and purity assays (> 94%) (Figures S7 & S8), mouse laminin 1 precoated transwells (Corning Costar, USA) were seeded with AT2 cells (10^6^ cells/cm^2^). Culture medium (DMEM/F12 + 2mM L-glutamine + 1% ITS + 1% BSA + 1× non-essential amino acids and 10 % new-born calf serum) was replaced with serum free media post 72 hours and then every 48 hours. Transepithelial resistance (R_T_) and potential difference (E_T_) were measured with an epithelial voltohmmeter (World Precision Instrument, USA). I_EQ_ values were calculated as the ratio of E_T_ / R_T_ values.

**Mouse tracheal epithelial cell isolations and monolayer cultures**. MTE cells were isolated from C57BL/6 and *Scnn1d* Tg/Cre mice and cultured as reported previously 73. Briefly, mice were anesthetized with intraperitoneal injection of ketamine HCl and xylazine. The trachea proximal to the bronchial bifurcation was isolated and removed. The resected section was immediately placed in PBS. Under a dissecting microscope, esophageal remnants and adherent adipose tissue were removed, and the tracheal sections were opened longitudinally, rinsed in PBS, and rotated in fresh DMEM containing 0.1% protease XIV, 0.01% DNase, and 1% FBS for 24 hours. Cells were pelleted by centrifugation, suspended twice in fresh DMEM containing 5% FBS, and seeded onto 6.5-mm diameter, collagen-coated transwell inserts (Corning-Costar, Lowell, MA) at a density of ∼3 × 10^5^ cells/cm^2^. MTE cells were grown in a 1:1 mixture of 3T3 fibroblast preconditioned DMEM (containing 10% FBS, 1% penicillin/streptomycin) and Ham's F-12 medium, supplemented with 10 μg/ml insulin, 1 μM hydrocortisone, 250 nM dexamethasone, 3.75 μg/ml endothelial cell growth supplement, 25 ng/ml epidermal growth factor, 30 nM triiodothyronine, 5 μg/ml iron saturated transferrin, and 10 ng/ml cholera toxin. The culture medium on the basolateral side of the filters was replaced every 48 hours. Apical medium was removed 4 days post seeding and then every time the basolateral culture medium was changed, and cells were cultured for 7-9 additional days at an air-liquid interface. Transepithelial resistance was monitored every other day when culture medium was replaced by use of an epithelial voltohmmeter (WPI, Sarasota, FL). Cell-growing inserts with a resistance >1,000 Ω were used.

**Organotypic cultures**. AT2 cells were cultured as organoids as previously described 74. Briefly, MLG-2908 cells (2 × 10^5^ cells/ml) were mixed with 6,000 AT2 cells and pelleted down. Cells were then resuspended into a 100 µl mixture (1:1) of growth factor reduced Matrigel (Corning, USA) and organoid media (DMEM/F12 + 2 mM L-glutamine + 10 % active FBS + 1% ITS and 10 μM TGF-β rock inhibitor). The mixed cells (90 μl) were seeded on the 0.4 µm inserts (Corning Costar, USA) and incubated for 30 minutes to allow the matrix to solidify, and 410 µl of medium was added to the bottom well. One half (200 µl) of the medium was changed every other day. After ~8 days, colonies (diameter ≥ 50 μm) were visualized and counted with either a Zeiss LSM510 microscope (Carl Zeiss AG, Germany) or an Olympus IX 73 microscope combined with a microscope objective (4×, NA:0.16, FN:26.5, UPlanSAPo, Olympus, Tokyo, Japan). DIC images of all the fields across transwells were captured with a Hamamatsu photonics CMOS Camera (Orca Flash 4.0; 2,048 × 2,048 pixels) and counted for total number of colonies. Surface area of the organoids was measured by marking area across the colonies and then measured with the ImageJ software (Figure S6). Cultured mouse organoids were incubated in PBS containing 4% paraformaldehyde for 20 minutes at room temperature for fixation. The fixed organoids were immunostained with AT1 and AT2 antibodies.

**Ussing chamber recordings**. Measurements of short-circuit current (*Isc*) in MTE monolayers were performed as described previously 73, 75. Briefly, MTE monolayers were mounted in vertical Ussing chambers (Physiologic Instruments, San Diego, CA) and bathed with solutions containing (in mM) 120 NaCl, 25 NaHCO_3_, 3.3 KH_2_PO_4_, 0.83 K_2_HPO_4_, 1.2 CaCl_2_, 1.2 MgCl_2_, 10 HEPES, 10 mannitol (apical compartment), or 10 D-glucose (basolateral compartment). Each solution was iso-osmolalic. The filters were bathed with the salt solution, bubbled continuously with a 95% O_2_ - 5% CO_2_ gas mixture (pH 7.4). The transmonolayer potential was short circuited to 0 mV, and *Isc* level was measured with an epithelial voltage clamp (VCC-MC8, Physiologic Instruments). A 10-mV pulse of 1-sec duration was imposed every 10 sec to monitor transepithelial resistance. Data were collected with the Acquire and Analyze program (version 2.3; Physiologic Instruments). When the *Isc* level reached a plateau, drugs were pipetted to the apical compartment.

**Cloning, *in vitro* transcription, and heterologous expression of δ_802_ ENaC and two-electrode voltage clamping assay**. δ_802_ and other ENaC subunits were generated in human ENaC cDNAs cloned into a pGEM HE vector 4, 76. cRNAs of human α, β, and γ ENaC were prepared as described previously^77^. The stoichiometry of native ENaC complexes is unclear to date. Based on the studies in the last decade, several models have been proposed for αβγ ENaC channels: 1α1β1γ, 2α1β1γ, 3α3β3γ. Based on the EM structure of human αβγ ENaC 35, the currently acceptable model of human αβγ ENaC complexes is heterotrimeric (1α1β1γ). Thus we microinjected δ802:β:γ in a 1:1:1 subunit ratio. Oocytes were surgically removed from appropriately anesthetized adult female *Xenopus laevis* (Xenopus Express). Briefly, the ovarian tissue was removed from the frog under anesthesia by tricaine-S (Western Chemicals) through a small incision in the lower abdomen. Ovarian lobes were removed and digested in OR-2 calcium-free medium (in mM: 82.5 NaCl, 2.5 KCl, 1.0 MgCl_2_, 1.0 Na_2_HPO_4_, and 10.0 HEPES, pH 7.5) with the addition of 2 mg/ml collagenase (Roche, Indianapolis). Defolliculated oocytes were injected with ENaC cRNAs into the cytosol (25 ng per oocyte in 50 nl of RNase-free water) and incubated in regular OR-2 medium at 18°C. The two-electrode voltage-clamp technique was used to record whole-cell currents 48 h post injection. Oocytes were impaled with two electrodes filled with 3M KCl, having resistance of 0.5 - 2 MΩ. A TEV-200A voltage-clamp amplifier (Dagan) was used to clamp oocytes with concomitant recording of currents. Two reference electrodes were connected to the bath. The continuously perfused bathing solution was ND-96 medium (in mM: 96.0 NaCl, 1.0 MgCl_2_, 1.8 CaCl_2_, 2.5 KCl, and 5.0 HEPES, pH 7.5). Whole-cell currents were recorded as previously reported 24. Experiments were controlled by pCLAMP 10.7 software (Molecular Devices), and currents at -40 mV, -100 mV, and +80 mV were continuously monitored with data recorded at intervals of 10 sec. Data were sampled at the rate of 200 Hz and filtered at 50 Hz.

**Establishment of humanized transgenic mouse strain conditionally expressing human δ_802_-ENaC**. HA and His-tagged human δ_802_-ENaC encoding sequence was constructed by extending the N-terminal of the δ2-ENaC clone and substituting few amino acid residues 29. The *hSCNN1D* construct was inserted into wild type ROSA26-EGFP vector. Transgenic mice on a C57BL/6/129Sv genetic background were generated by aggregation of targeted ES-cells with eight-cell stage embryos by the Biocytogen (Boston Co. USA) without marked phenotype of emphysema (Figures S4 & S9). Germline transmitting mice were genotyped by Southern blotting and PCR for the transmission of the transgene. Conditional induction of δ_802_ ENaC expression was carried out by cross bred with sox2^Cre^ (Jackson Laboratory, 004682) and SPC^Cre^ (Jackson Laboratory, cat.#004682).

**Surface biotinylation and immunoblotting assays**. Primary MTE and AT2 cells grown on permeable transwell inserts were incubated with cell-impermeant sulfo-NHS-SS-biotin (1 mg/ml, Pierce, cat.#21331) in PBS (pH 8.0) for 30 minutes. Biotinylated cells were harvested and lysed in lysis buffer (Cell Signalling, Danvers, MA). The biotinylated membrane proteins were purified with Pierce High Capacity NeutrAvidin Agarose (Pierce, cat.#29202). Quantified proteins were probed with specific monoclonal anti-HA antibody. The blots were stripped and re-probed with an antibody against β-actin (as a loading control of cytosolic proteins). Protein signals were detected by chemiluminescence (Millipore) with Genemate Blue Light Film (ISC).

**Confocal imaging of organoids**. Proliferation and differentiation of AT2 cells in 3D cultures on day 7 were determined by immunostaining with rabbit sftpc (EMD Millipore, USA) and Syrian Hamster pdpn (Thermo Fisher Scientific, USA). Primary antibodies were detected by anti-rabbit Alexa Flour488, anti-Syrian Hamster Alexa Flour568 (Thermo Fisher Scientific, USA). Fluorescence images were obtained using a Zeiss LSM 510 confocal microscope and stacked with the Fiji component of the ImageJ. Monolayers and organoids were scanned for Z sections with optimal width from top to bottom. Continuous images were stacked separately for pdpn and sftpc to count the number of positive cells for both by using plugin for cell counter in the Image J. Both pdpn^+^ and sftpc^+^ cells were counted and analysed statistically. Each slide was scanned for 6 different fields (n = 3 different experiments). For three-dimensional reconstruction, a series of optical sections that were obtained by confocal microscope were collected at 0.5-μm intervals moving progressively across the cells. For 3D structure, all Z sections were stacked from top to bottom and saved as AVI files.

**FACS assays of differentiation and proliferation**. For analysis of AT2 cells proliferation and differentiation, AT2 organoids from different experimental groups were dissociated from Matrigel with dispase (10 units/ml) and digested in 0.25% trypsin-EDTA to get single cells suspension. Cells were then stained with antibodies purchased from Biolegend (AF488-EpCAM, APC-ICAM and APC-PDPN). Gates for both colours were set by unstained cells and isotype controls for each antibody. We have used a strategy to use double colour staining (AF488-EpCAM, APC-ICAM) to enhance the separation of AT1 and AT2 cells in organoid suspension. Cells were analysed by the FACSCaliber^TM^ (BD, USA) and the results were analysed using the FlowJow 10.1 software.

**Immunohistology of lung tissues and visualization of AT1 and AT2 cells**. Lung tissue sections were first deparaffinized and subjected to antigen retrieval using a citrate buffer at 95°C for 20 minutes. Immunostaining was performed by using antibodies of anti-pro-SPC (1:1000, Thermo Fisher Scientific), anti-AQP5 (1:500, EMD Millipore), and anti-His (1:1000, Abcam). Mouse tissue sections were blocked using a proprietary blocking solution from a M.O.M. kit (Vector Laboratories). The primary antibodies were then incubated overnight at 4°C in the diluent solution provided by the kit, and were visualized with Alexa Fluor 488, 568, and 647 secondary antibodies (Life Technologies). Nuclei were stained with Hoechst 33342 (Thermo Fisher Scientific). Tissue slices were mounted onto slides with ProLong Gold Antifade Reagent (Life Technologies). Tissue staining images were taken by Zeiss LSM 510 confocal systems (Zeiss) for immunofluorescence staining. After fixation, organoids were dehydrated with increasing ethanol grades and embedded as paraffin blocks. Seven-μm thick sections were cut and fixed for additional 5 minutes in 4% PFA at room temperature and stained with a routine H & E protocol.

**EdU assay for DNA synthesis**. AT2 monolayer cells with active DNA synthesis were detected with Click-iT^TM^ EdU assay kit. Cell monolayers from both wt and *Scnn1d*^Tg/cre^ groups were stained with the Click-iT^TM^ EdU alexa flour 488 following the manufacturer's instructions. Images were captured and analyzed for the percentage of EdU^+^ cells in different experimental groups. Ten randomly selected images across the monolayers from at least 3 independent experiments were captured and counted for total cells and EdU^+^ portion using a Cell Count plug-in of the ImageJ. The percentage of EdU^+^ cells was calculated for each group, and the difference among groups was compared statistically as described below.

**Quantitative measurements of two δENaC variants and full-length clones**. Total RNA was extracted from human lung tissues use Qiagen RNeasy Plus Mini Kit (cat.# 74136), according to the manufacturer's instructions, and RNA concentration was measured by spectrophotometry at 260 nm. An iScript reverse transcription Supermix for RT-qPCR was used to synthesize cDNA (Bio-Rad, cat.# 1708841). The following primer pairs synthesized by Sigma-Aldrich were used for real-time PCR. δ1-ENaC: 5'-CTC TCA CCT CCA GGC TGC-3' (forward) and 5'-GGC ATT GGT GCA GAA GAA GG-3' (reverse); δ2-ENaC: 5'-CCC TGC CAC CTG AAG GGA TG-3' (forward) and 5'-ATT GGT GCA GAA GAA GGT GAG C-3' (reverse); δ_802_-ENaC: 5'-AAG ACA ACA CCG CTC CCT CG-3' (forward) and 5'-TGT GGG GGT CCT GTG GTC A-3' (reverse); human GAPDH: 5'- GGA GTC AAC GGA TTT GGT CGT-3' (forward) and 5'-CCC GTT CTC AGC CAT GTA GTT G-3' (reverse). qPCR assays were performed with a SsoAdvanced Universal SYBR Green Supermix (Bio-Rad cat.# 1725271). Reaction for δ-ENaC variants and GAPDH was performed using a single cycle of 95°C for 1 minute, followed by 40 cycles of 95°C for 10 s and 58°C 30 s. Relative expression of δ-ENaC variants was calculated using the 2^Δct^ comparative method, with normalization of each sample against the expression of endogenous reference gene GAPDH.

**Statistical analysis**. All data are presented as the mean ± s.e.m. for n experiments. Differences between means were tested for statistical significance using paired or unpaired t-tests or their non-parametric equivalents as appropriate. Differences between groups were judged using analysis of variance. From such comparisons, differences yielding P < 0.05 were judged to be significant. Either GraphPad Prism or OriginLab was used for statistical analysis and graphing.

## Supplementary Material

Supplementary figures and tables.

## Abbreviations

ENaC: epithelial sodium channels
DEG): degenerins
tc-uPA): Two-chain urokinase plasminogen activator
MTE): mouse tracheal epithelial
AT2: type 2 alveolar epithelial cells
AT1: type 1 alveolar epithelial cells
Isc: short-circuit current
pdpn: podoplanin
sftpc: surfactant protein C
ICAM: intercellular adhesion molecule 1
EpCAM: epithelial cellular adhesion molecule
EdU: 5-ethynyl-2´-deoxyuridine
Scnn1d: sodium channel nonvoltage-gated 1, delta subunit
FaNaC: FMRFamide-gated sodium channel
PPK: pickpocket
RPK: ripped pocket
BLINaC: brain liver intestinal sodium channel
ASIC: acid-sensing ion channel
CFTR: cystic fibrosis transmembrane conductance regulator
AFC: alveolar fluid clearance
