# Supplementary material for: Proliferative regulation of alveolar epithelial type 2 progenitor cells by human Scnn1d gene
Source: Theranostics. 2019 Oct 18;9(26):8155–70. doi: 10.7150/thno.37023 (PMC6857051; doi:10.7150/thno.37023)

Figure S1. Alignments of δ_{802} and δ_2 human ENaC variants.

1) Nucleotide alignment of human δ_{802} and δ_2 ENaC variants.

```
DQ898176.1_704_         ------------------------------------------------------------
NM_001130413.3_802_     ATGAGGGCAGTGCTGTCACAGAAGACAACACCGCTCCCTCGTTACCTGTGGCCCGGCCAC 60


DQ898176.1_704_         ------------------------------------------------------------
NM_001130413.3_802_     CTCAGCGGCCCAAGGAGGCTCACCTGGTCATGGTGCAGTGACCACAGGACCCCCACATGC 120


DQ898176.1_704_         ------------------------------------------------------------
NM_001130413.3_802_     CGGGAGCTGGGTTCGCCCCACCCCACCCCCTGCACCGGGCCAGCGAGGGGATGGCCCAGA 180


DQ898176.1_704_         ------------------------------------------------------------
NM_001130413.3_802_     AGAGGGGGAGGACCATGTGGATTCACCAGTGCTGGACATGTGCTCTGTGGCTACCCCCTC 240


DQ898176.1_704_         ------------------------------------------------------ATGGCT 6
NM_001130413.3_802_     TGCCTACTCTCTGGCCCGATACAGGGGTGTGGGACAGGCCTGGGTGACTCCAGCATGGCT 300
                                                                              ******

DQ898176.1_704_         TTCCTCTCCAGGACGTCACCGGTGGCAGCTGCTTCCTTCCAGAGCCGGCAGGAGGCCAGA 66
NM_001130413.3_802_     TTCCTCTCCAGGACGTCACCGGTGGCAGCTGCTTCCTTCCAGAGCCGGCAGGAGGCCAGA 360
                        ************************************************************

DQ898176.1_704_         GGCTCCATCCTGCTTCAGAGCTGCCAGCTGCCCCCGCAATGGCTGAGCACCGAAGCATGG 126
NM_001130413.3_802_     GGCTCCATCCTGCTTCAGAGCTGCCAGCTGCCCCCGCAATGGCTGAGCACCGAAGCATGG 420
                        ************************************************************

DQ898176.1_704_         ACGGGGGAATGGAAGCAGCCACACGGGGGGGCTCTCACCTCCAGATCGCCTGGGCCTGTG 186
NM_001130413.3_802_     ACGGGAGAATGGAAGCAGCCACACGGGGGGGCTCTCACCTCCAGATCGCCTGGGCCTGTG 480
```

```
                           *****.******************************************************

DQ898176.1_704_            GCTCCCCAGAGGCCCTGCCACCTGAAGGGATGGCAGCACAGACCCGCTCAGCACAACGCT 246
NM_001130413.3_802_        GCTCCCCAGAGGCCCTGCCACCTGAAGGGATGGCAGCACAGACCCACTCAGCACAACGCT 540
                           *********************************************.**************

DQ898176.1_704_            GCCTGCAAACAGGGCCAGGCTGCAGCCCAGACGCCCCCCAGGCCGGGGCCACCATCAGCA 306
NM_001130413.3_802_        GCCTGCAAACAGGGCCAGGCTGCAGCCCAGACGCCCCCCAGGCCGGGGCCACCATCAGCA 600
                           ************************************************************

DQ898176.1_704_            CCACCACCACCACCCAAGGAGGGGCACCGGGAGGGGCTGGTGGAGCTGCCCGCCTCGTTC 366
NM_001130413.3_802_        CCACCACCACCACCCAAGGAGGGGCACCAGGAGGGGCTGGTGGAGCTGCCCGCCTCGTTC 660
                           ****************************.*******************************

DQ898176.1_704_            CGGGAGCTGCTCACCTTCTTCTGCACCAATGCCACCATCCACGGCGCCATCCGCCTGGTC 426
NM_001130413.3_802_        CGGGAGCTGCTCACCTTCTTCTGCACCAATGCCACCATCCACGGCGCCATCCGCCTGGTC 720
                           ************************************************************

DQ898176.1_704_            TGCTCCCGCGGGAACCGCCTCAAGACGACGTCCTGGGGGCTGCTGTCCCTGGGAGCCCTG 486
NM_001130413.3_802_        TGCTCCCGCGGGAACCGCCTCAAGACGACGTCCTGGGGGCTGCTGTCCCTGGGAGCCCTG 780
                           ************************************************************

DQ898176.1_704_            GTCGCGCTCTGCTGGCAGCTGGGGCTCCTCTTTGAGCGTCACTGGCACCGCCCGGTCCTC 546
NM_001130413.3_802_        GTCGCGCTCTGCTGGCAGCTGGGGCTCCTCTTTGAGCGTCACTGGCACCGCCCGGTCCTC 840
                           ************************************************************

DQ898176.1_704_            ATGGCCGTCTCTGTGCACTCGGAGCGCAAGCTGCTCCCGCTGGTCACCCTGTGTGACGGG 606
NM_001130413.3_802_        ATGGCCGTCTCTGTGCACTCGGAGCGCAAGCTGCTCCCGCTGGTCACCCTGTGTGACGGG 900
                           ************************************************************

DQ898176.1_704_            AACCCACGTCGGCCGAGTCCGGTCCTCCGCCATCTGGAGCTGCTGGACGAGTTTGCCAGG 666
NM_001130413.3_802_        AACCCACGTCGGCCGAGTCCGGTCCTCCGCCATCTGGAGCTGCTGGACGAGTTTGCCAGG 960
                           ************************************************************
```

```
DQ898176.1_704_          GAGAACATTGACTCCCTGTACAACGTCAACCTCAGCAAAGGCAGAGCCGCCCTCTCCGCC 726
NM_001130413.3_802_      GAGAACATTGACTCCCTGTACAACGTCAACCTCAGCAAAGGCAGAGCCGCCCTCTCCGCC
1020
                         ************************************************************

DQ898176.1_704_          ACTGTCCCCCGCCACGAGCCCCCCTTCCACCTGGACCGGGAGATCCGTCTGCAGAGGCTG 786
NM_001130413.3_802_      ACTGTCCCCCGCCACGAGCCCCCCTTCCACCTGGACCGGGAGATCCGTCTGCAGAGGCTG
1080
                         ************************************************************

DQ898176.1_704_          AGCCACTCGGGCAGCCGGGTCAGAGTGGGGTTCAGACTGTGCAACAGCACGGGCGGCGAC 846
NM_001130413.3_802_      AGCCACTCGGGCAGCCGGGTCAGAGTGGGGTTCAGACTGTGCAACAGCACGGGCGGCGAC
1140
                         ************************************************************

DQ898176.1_704_          TGCTTTTACCGAGGCTACACGTCAGGCGTGGCGGCTGTCCAGGACTGGTACCACTTCCAC 906
NM_001130413.3_802_      TGCTTTTACCGAGGCTACACGTCAGGCGTGGCGGCTGTCCAGGACTGGTACCACTTCCAC
1200
                         ************************************************************

DQ898176.1_704_          TATGTGGATATCCTGGCCCTGCTGCCCGCGGCATGGGAGGACAGCCACGGGAGCCAGGAC 966
NM_001130413.3_802_      TATGTGGATATCCTGGCCCTGCTGCCCGCGGCATGGGAGGACAGCCACGGGAGCCAGGAC
1260
                         ************************************************************

DQ898176.1_704_          GGCCACTTCGCCCTCTCCTGCAGTTACGATGGCCTGGACTGCCAGGCCCGACAGTTCCGG
1026
NM_001130413.3_802_      GGCCACTTCGTCCTCTCCTGCAGTTACGATGGCCTGGACTGCCAGGCCCGACAGTTCCGG
1320
                         ********** *************************************************

DQ898176.1_704_          ACCTTCCACCACCCCACCTACGGCAGCTGCTACACGGTCGATGGCGTCTGGACAGCTCAG
1086
```

NM_001130413.3_802_ ACCTTCCACCACCCCACCTACGGCAGCTGCTACACGGTCGATGGCGTCTGGACAGCTCAG
1380
****

DQ898176.1_704_ CGCCCCGGCATCACCCACGGAGTCGGCCTGGTCCTCAGGGTTGAGCAGCAGCCTCACCTC
1146
NM_001130413.3_802_ CGCCCCGGCATCACCCACGGAGTCGGCCTGGTCCTCAGGGTTGAGCAGCAGCCTCACCTC
1440
****

DQ898176.1_704_ CCTCTGCTGTCCACGCTGGCCGGCATCAGGGTCATGGTTCACGGCCGTAACCACACGCCC
1206
NM_001130413.3_802_ CCTCTGCTGTCCACGCTGGCCGGCATCAGGGTCATGGTTCACGGCCGTAACCACACGCCC
1500
****

DQ898176.1_704_ TTCCTGGGGCACCACAGCTTCAGCGTCCGGCCAGGGACGGAGGCCACCATCAGCATCCGA
1266
NM_001130413.3_802_ TTCCTGGGGCACCACAGCTTCAGCGTCCGGCCAGGGACGGAGGCCACCATCAGCATCCGA
1560
****

DQ898176.1_704_ GAGGACGAGGTGCACCGGCTCGGGAGCCCCTACGGCCACTGCACCGCCGGCGGGGAAGGC
1326
NM_001130413.3_802_ GAGGACGAGGTGCACCGGCTCGGGAGCCCCTACGGCCACTGCACCGCCGGCGGGGAAGGC
1620
****

DQ898176.1_704_ GTGGAGGTGGAGCTGCTACACAACACCTCCTACACCAGGCAGGCCTGCCTGGTGTCCTGC
1386
NM_001130413.3_802_ GTGGAGGTGGAGCTGCTACACAACACCTCCTACACCAGGCAGGCCTGCCTGGTGTCCTGC
1680
****

```
DQ898176.1_704_         TTCCAGCAGCTGATGGTGGAGACCTGCTCCTGTGGCTACTACCTCCACCCTCTGCCGGCG
1446
NM_001130413.3_802_     TTCCAGCAGCTGATGGTGGAGACCTGCTCCTGTGGCTACTACCTCCACCCTCTGCCGGCG
1740
                        ************************************************************

DQ898176.1_704_         GGGGCTGAGTACTGCAGCTCTGCCCGGCACCCTGCCTGGGGACACTGCTTCTACCGCCTC
1506
NM_001130413.3_802_     GGGGCTGAGTACTGCAGCTCTGCCCGGCACCCTGCCTGGGGACACTGCTTCTACCGCCTC
1800
                        ************************************************************

DQ898176.1_704_         TACCAGGACCTGGAGACCCACCGGCTCCCCTGTACCTCCCGCTGCCCCAGGCCCTGCAGG
1566
NM_001130413.3_802_     TACCAGGACCTGGAGACCCACCGGCTCCCCTGTACCTCCCGCTGCCCCAGGCCCTGCAGG
1860
                        ************************************************************

DQ898176.1_704_         GAGTCTGCATTCAAGCTCTCCACTGGGACCTCCAGGTGGCCTTCCGCCAAGTCAGCTGGA
1626
NM_001130413.3_802_     GAGTCTGCATTCAAGCTCTCCACTGGGACCTCCAGGTGGCCTTCCGCCAAGTCAGCTGGA
1920
                        ************************************************************

DQ898176.1_704_         TGGACTCTGGCCACGCTAGGTGAACAGGGGCTGCCGCATCAGAGCCACAGACAGAGGAGC
1686
NM_001130413.3_802_     TGGACTCTGGCCACGCTAGGTGAACAGGGGCTGCCGCATCAGAGCCACAGACAGAGGAGC
1980
                        ************************************************************

DQ898176.1_704_         AGCCTGGCCAAAATCAACATCGTCTACCAGGAGCTCAACTACCGCTCAGTGGAGGAGGCG
1746
NM_001130413.3_802_     AGCCTGGCCAAAATCAACATCGTCTACCAGGAGCTCAACTACCGCTCAGTGGAGGAGGCG
2040
```

```
                    ************************************************************

DQ898176.1_704_     CCCGTGTACTCGGTGCCGCAGCTGCTCTCGGCCATGGGCAGCCTCTGCAGCCTGTGGTTT
1806
NM_001130413.3_802_ CCCGTGTACTCGGTGCCGCAGCTGCTCTCGGCCATGGGCAGCCTCTGCAGCCTGTGGTTT
2100
                    ************************************************************

DQ898176.1_704_     GGGGCCTCCGTCCTCTCCCTCCTGGAGCTCCTGGAGCTGCTGCTCGATGCTTCTGCCCTC
1866
NM_001130413.3_802_ GGGGCCTCCGTCCTCTCCCTCCTGGAGCTCCTGGAGCTGCTGCTCGATGCTTCTGCCCTC
2160
                    ************************************************************

DQ898176.1_704_     ACCCTGGTGCTAGGCGGCCGCCGGCTCCGCAGGGCGTGGTTCTCCTGGCCCAGAGCCAGC
1926
NM_001130413.3_802_ ACCCTGGTGCTAGGCGGCCGCCGGCTCCGCAGGGCGTGGTTCTCCTGGCCCAGAGCCAGC
2220
                    ************************************************************

DQ898176.1_704_     CCTGCCTCAGGGGCGTCCAGCATCAAGCCAGAGGCCAGTCAGATGCCCCCGCCTGCAGGC
1986
NM_001130413.3_802_ CCTGCCTCAGGGGCGTCCAGCATCAAGCCAGAGGCCAGTCAGATGCCCCCGCCTGCAGGC
2280
                    ************************************************************

DQ898176.1_704_     GGCACGTCAGATGACCCGGAGCCCAGCGGGCCTCATCTCCCACGGGTGATGCTTCCAGGG
2046
NM_001130413.3_802_ GGCACGTCAGATGACCCGGAGCCCAGCGGGCCTCATCTCCCACGGGTGATGCTTCCAGGG
2340
                    ************************************************************

DQ898176.1_704_     GTTCTGGCGGGAGTCTCAGCCGAAGAGAGCTGGGCTGGGCCCCAGCCCCTTGAGACTCTG
2106
```

```
NM_001130413.3_802_      GTTCTGGCGGGAGTCTCAGCCGAAGAGAGCTGGGCTGGGCCCCAGCCCCTTGAGACTCTG
2400
                         ************************************************************

DQ898176.1_704_          GACACCTGA 2115
NM_001130413.3_802_      GACACCTGA 2409
                         *********
```

2) Amino acid sequence alignment of human δ_{802} and δ_2 human ENaC variants.

```
NP_001123885.2_802_      MRAVLSQKTTPLPRYLWPGHLSGPRRLTWSWCSDHRTPTCRELGSPHPTPCTGPARGWPR 60
ABI64069.1      704      ------------------------------------------------------------

NP_001123885.2_802_      RGGGPCGFTSAGHVLCGYPLCLLSGPIQGCGTGLGDSSMAFLSRTSPVAAASFQSRQEAR 120
ABI64069.1               --------------------------------------MAFLSRTSPVAAASFQSRQEAR 22
                                                               **********************

NP_001123885.2_802_      GSILLQSCQLPPQWLSTEAWTGEWKQPHGGALTSRSPGPVAPQRPCHLKGWQHRPTQHNA 180
ABI64069.1               GSILLQSCQLPPQWLSTEAWTGEWKQPHGGALTSRSPGPVAPQRPCHLKGWQHRPAQHNA 82
                         *******************************************************:****

NP_001123885.2_802_      ACKQGQAAAQTPPRPGPPSAPPPPPKEGHQEGLVELPASFRELLTFFCTNATIHGAIRLV 240
ABI64069.1               ACKQGQAAAQTPPRPGPPSAPPPPPKEGHREGLVELPASFRELLTFFCTNATIHGAIRLV 142
                         *****************************:******************************
                                                       M1
NP_001123885.2_802_      CSRGNRLKTTSWGLLSLGALVALCWQLGLLFERHWHRPVLMAVSVHSERKLLPLVTLCDG 300
ABI64069.1               CSRGNRLKTTSWGLLSLGALVALCWQLGLLFERHWHRPVLMAVSVHSERKLLPLVTLCDG 202
                         ************************************************************

NP_001123885.2_802_      NPRRPSPVLRHLELLDEFARENIDSLYNVNLSKGRAALSATVPRHEPPFHLDREIRLQRL 360
ABI64069.1               NPRRPSPVLRHLELLDEFARENIDSLYNVNLSKGRAALSATVPRHEPPFHLDREIRLQRL 262
                         ************************************************************

NP_001123885.2_802_      SHSGSRVRVGFRLCNSTGGDCFYRGYTSGVAAVQDWYHFHYVDILALLPAAWEDSHGSQD 420
ABI64069.1               SHSGSRVRVGFRLCNSTGGDCFYRGYTSGVAAVQDWYHFHYVDILALLPAAWEDSHGSQD 322
                         ************************************************************

NP_001123885.2_802_      GHFVLSCSYDGLDCQARQFRTFHHPTYGSCYTVDGVWTAQRPGITHGVGLVLRVEQQPHL 480
ABI64069.1               GHFALSCSYDGLDCQARQFRTFHHPTYGSCYTVDGVWTAQRPGITHGVGLVLRVEQQPHL 382
                         ***.********************************************************
```

```
NP_001123885.2_802_      PLLSTLAGIRVMVHGRNHTPFLGHHSFSVRPGTEATISIREDEVHRLGSPYGHCTAGGEG 540
ABI64069.1               PLLSTLAGIRVMVHGRNHTPFLGHHSFSVRPGTEATISIREDEVHRLGSPYGHCTAGGEG 442
                         ************************************************************

NP_001123885.2_802_      VEVELLHNTSYTRQACLVSCFQQLMVETCSCGYYLHPLPAGAEYCSSARHPAWGHCFYRL 600
ABI64069.1               VEVELLHNTSYTRQACLVSCFQQLMVETCSCGYYLHPLPAGAEYCSSARHPAWGHCFYRL 502
                         ************************************************************

NP_001123885.2_802_      YQDLETHRLPCTSRCPRPCRESAFKLSTGTSRWPSAKSAGWTLATLGEQGLPHQSHRQRS 660
ABI64069.1               YQDLETHRLPCTSRCPRPCRESAFKLSTGTSRWPSAKSAGWTLATLGEQGLPHQSHRQRS 562
                         ************************************************************
                                                                   M2
NP_001123885.2_802_      SLAKINIVYQELNYRSVEEAPVYSVPQLLSAMGSLCSLWFGASVLSLLELLELLLDASAL 720
ABI64069.1               SLAKINIVYQELNYRSVEEAPVYSVPQLLSAMGSLCSLWFGASVLSLLELLELLLDASAL 622
                         ************************************************************

NP_001123885.2_802_      TLVLGGRRLRRAWFSWPRASPASGASSIKPEASQMPPPAGGTSDDPEPSGPHLPRVMLPG 780
ABI64069.1               TLVLGGRRLRRAWFSWPRASPASGASSIKPEASQMPPPAGGTSDDPEPSGPHLPRVMLPG 682
                         ************************************************************

NP_001123885.2_802_      VLAGVSAEESWAGPQPLETLDT 802
ABI64069.1               VLAGVSAEESWAGPQPLETLDT 704
                         **********************
```

Figure S2. Amino acid sequence of human α-13 and δ-15 peptides.

1) Human α-13 peptide, C-terminal amidation, N-terminal acetylation. "LRGTLPHPLQRLR".

2) Human δ-15 peptide, C-terminal amidation, N-terminal acetylation. "LSATVPRHEPPFHLD".

Figure S3. Confirmation of conditional expression by cross breeding CD19^{Cre} and hscnn1d^{Tg} lines. To confirm the conditional expression of human δ_{802} construct, the Biocytogen (http://www.biocytogen.com) bred $\delta_{802}{}^{Tg}$ with CD19^{Cre} and detected GFP^{+} cells in spleen and blood by FACS.

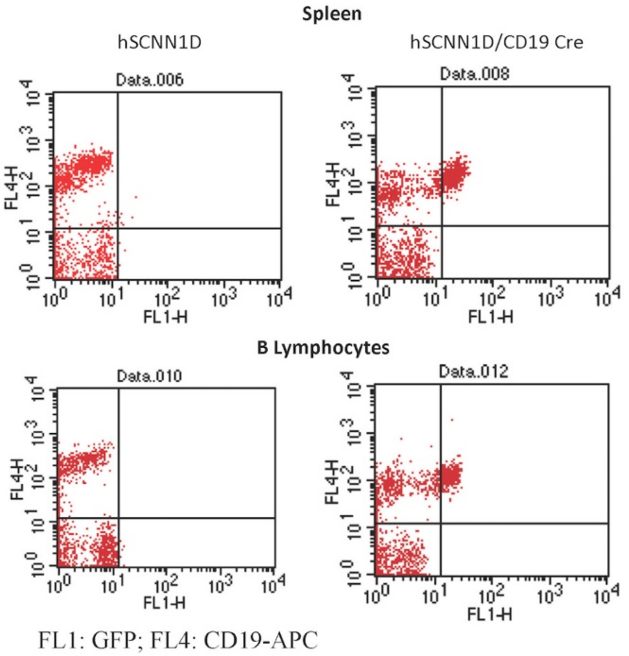

Spleen	Abs	Spleen
hSCNN1D	CD19+/GFP+	0.46%
hSCNN1D/CD19-Cre	CD19+/GFP+	31.60%

Blood	Abs	Spleen
hSCNN1D	CD19+/GFP+	0.02%
hSCNN1D/CD19-Cre	CD19+/GFP+	24.54%

Figure S4. Mass spectrometry analysis of two δ802 ENaC bands. AT2 cell lysates were immnoprecipitated with anti-HA antibody and immunoblotted with anti-his antibody. Then the blot was stained with Coomassie blue. Two bands near 100 kDa (selected by dashed line) were dissected for mass spectrometry. Corresponding MS1 spectra were shown on the right side. Both bands contained a human δENaC-specific peptide sequence (HLELLDEFAR).

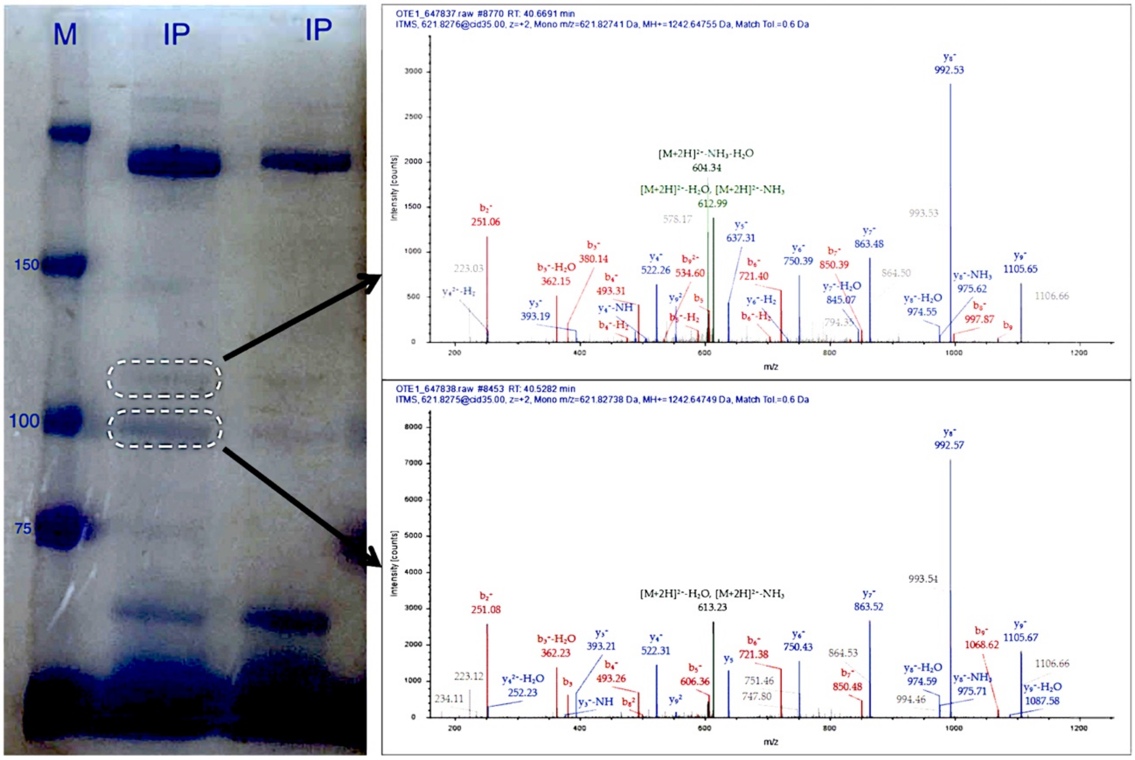

Figure S5. Visualization of tight junction of mouse AT2 monolayers. Image of mouse AT2 monolayers 5 days post seeding with anti-claudin 8 antibody. Scale bar, 100 μm.

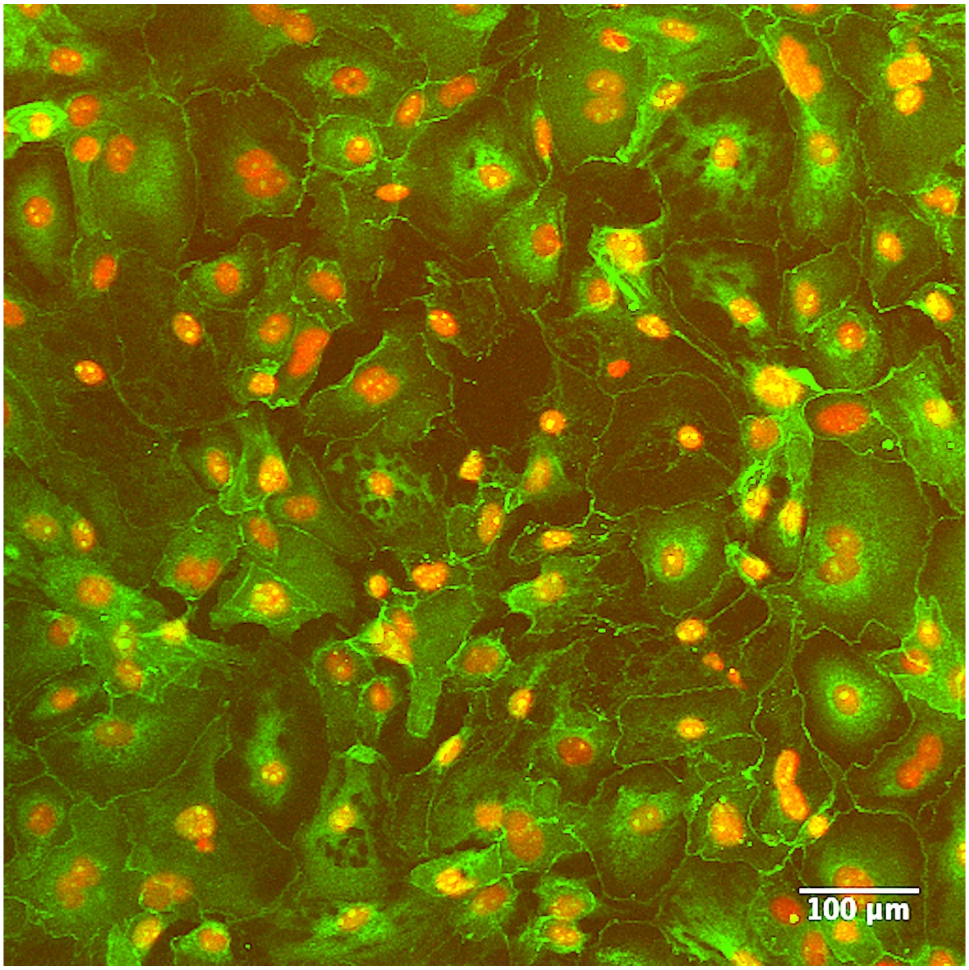

Figure S6. Images of an alveolus-like organoid formed by 3D cultured primary mouse AT2 cells. The organoid grown in 3D Matrigel for 12 days was imaged following stained with pdpn and sftpc antibodies. Dash lines, sideview level. Scale bar, 50 μm.

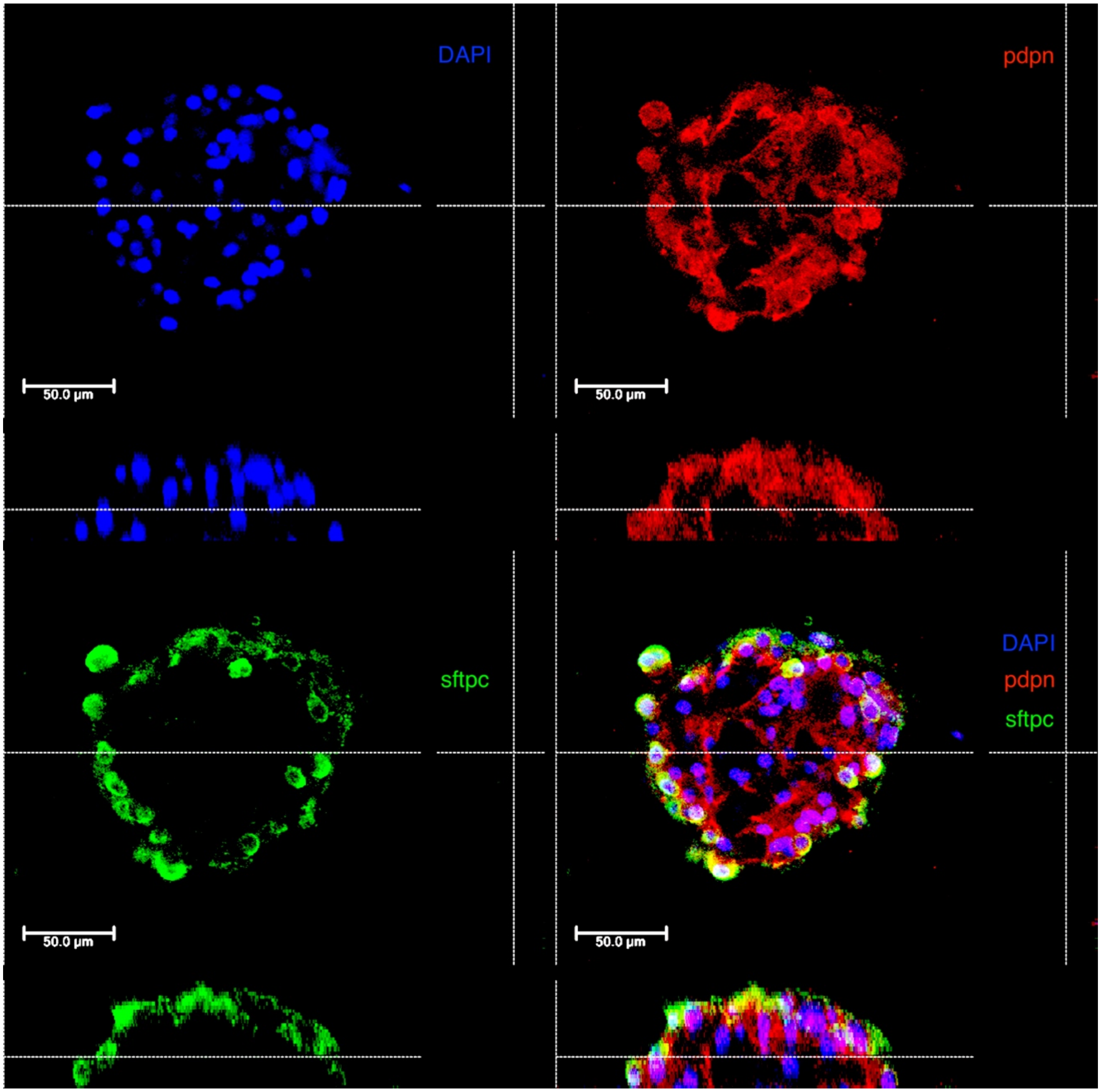

Figure S7. Image of isolated mouse AT2 cells. Top, freshly isolated mouse AT2 cells were stained with DAPI and anti-human pro-SPC antibody (sftpc) following cytospining. IgG without primary antibody was used for negative control. Scale bar, 50 μm. Bottom, PAP stain of freshly isolated mouse AT2 cells. Scale bar, 20 μm.

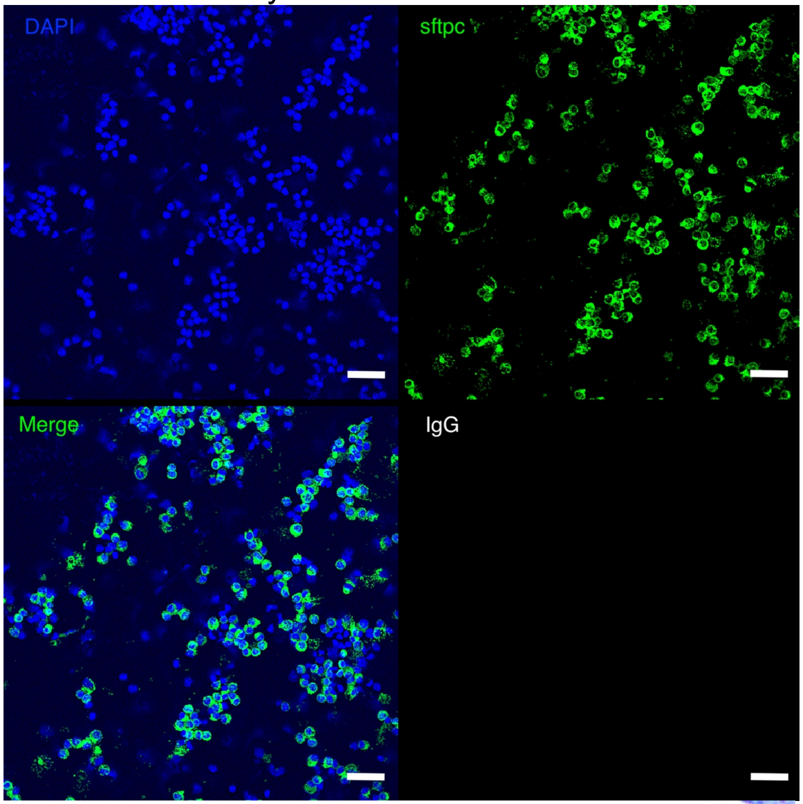

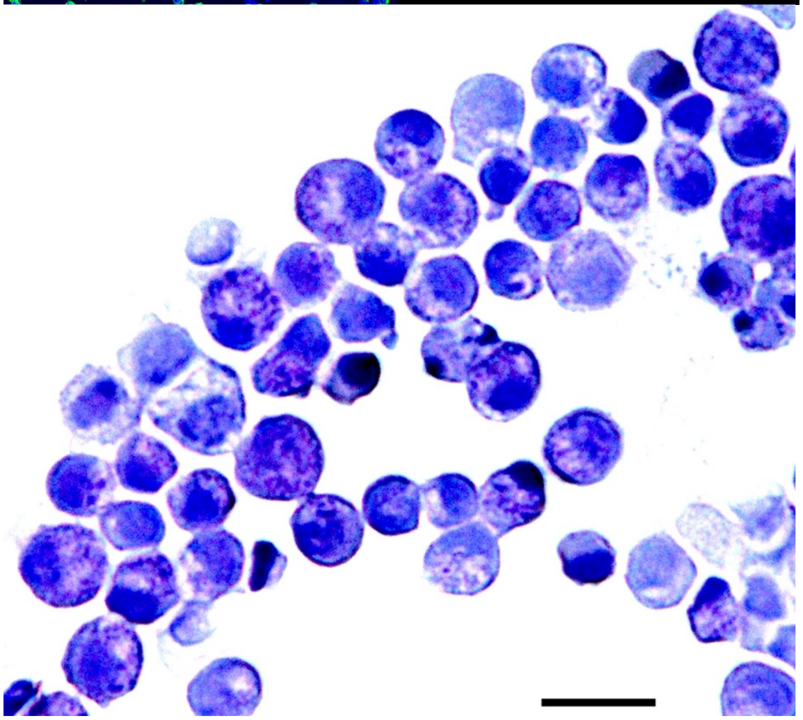

Figure S8. AT2 cells purity analyzed by FACS. Freshly isolated mouse AT2 cells were stained with EpCAM antibody (CD326). The purity is 96.58% (95 - 98%). n=8.

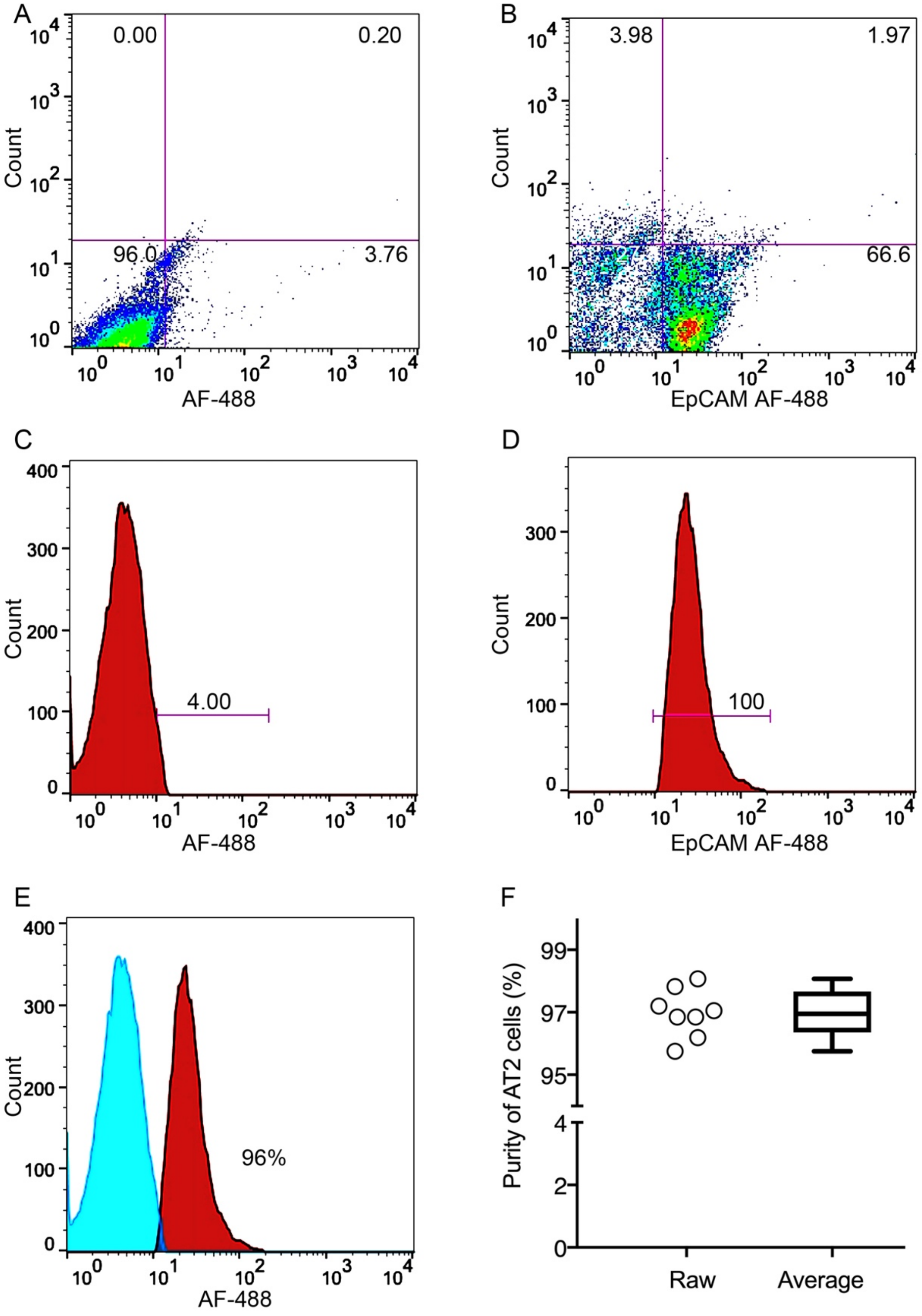

Figure S9. Histology of mouse lungs. Images of H & E stain of lung sections for wild type (wt) and human δ_{802}^{Tg} mice (Scnn1d Tg/sox2).

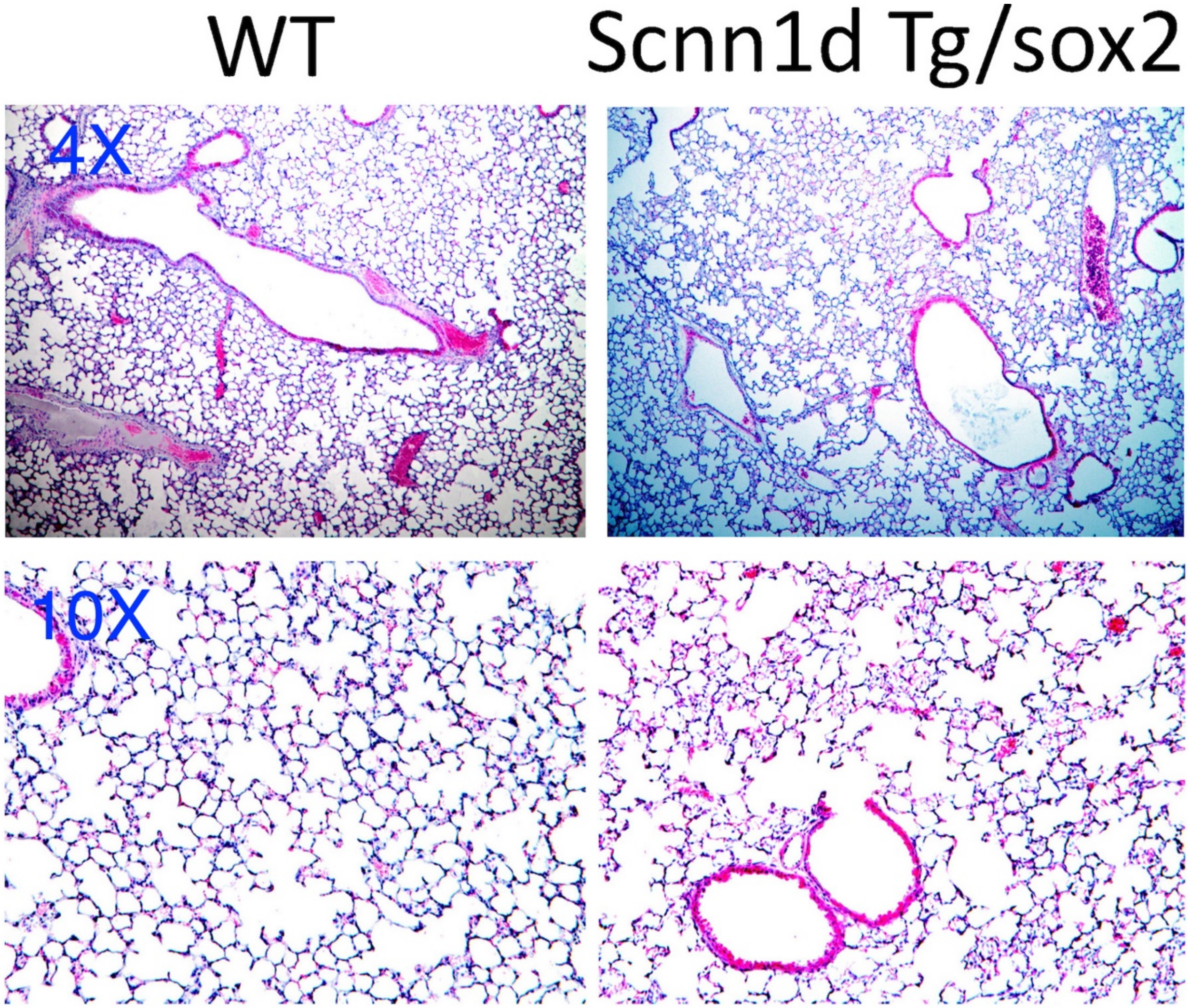

Supplement: Supplementary file 1 — Supplementary figures and tables. [file thnov09p8155s1.pdf]
